# Urinary tract infections in children: building a causal model-based decision support tool for diagnosis with domain knowledge and prospective data

**DOI:** 10.1186/s12874-022-01695-6

**Published:** 2022-08-08

**Authors:** Jessica A. Ramsay, Steven Mascaro, Anita J. Campbell, David A. Foley, Ariel O. Mace, Paul Ingram, Meredith L. Borland, Christopher C. Blyth, Nicholas G. Larkins, Tim Robertson, Phoebe C. M. Williams, Thomas L. Snelling, Yue Wu

**Affiliations:** 1 Wesfarmers Centre of Vaccines and Infectious Diseases, Telethon Kids Institute, University of Western Australia, Nedlands, WA 6009 Australia; 2 Bayesian Intelligence Pty Ltd, Upwey, VIC 3158 Australia; 3 Faculty of Information Technology, Monash University, Clayton, VIC 3168 Australia; 4 Department of Infectious Diseases, Perth Children’s Hospital, Nedlands, WA 6009 Australia; 5 Department of Microbiology, PathWest Laboratory Medicine, Nedlands, WA 6009 Australia; 6 Department of General Paediatrics, Perth Children’s Hospital, Nedlands, WA 6009 Australia; 7 School of Pathology and Laboratory Medicine, University of Western Australia, Nedlands, WA 6009 Australia; 8 Emergency Department, Perth Children’s Hospital, Nedlands, WA 6009 Australia; 9 Divisions of Emergency Medicine and Paediatrics, School of Medicine, University of Western Australia, Nedlands, WA 6009 Australia; 10 Faculty of Health and Medical Sciences, University of Western Australia, Crawley, Australia; 11 Department of Nephrology, Perth Children’s Hospital, Nedlands, WA 6009 Australia; 12 Child and Adolescent Health Service, Perth Children’s Hospital, Nedlands, WA 6009 Australia; 13 Sydney School of Public Health, Faculty of Medicine and Health, University of Sydney, 2006, Camperdown, NSW Australia; 14 Sydney Children’s Hospital Network, Randwick, NSW 2031 Australia; 15 School of Women’s and Children’s Health, The University of New South Wales, Sydney, NSW 2052 Australia; 16 School of Public Health, Curtin University, Bentley, WA 6102 Australia; 17 Menzies School of Health Research, Charles Darwin University, Darwin, NT 0815 Australia

**Keywords:** DAG, Causal model, Bayesian network, Clinical decision support, Urinary tract infection

## Abstract

**Background:**

Diagnosing urinary tract infections (UTIs) in children in the emergency department (ED) is challenging due to the variable clinical presentations and difficulties in obtaining a urine sample free from contamination. Clinicians need to weigh a range of observations to make timely diagnostic and management decisions, a difficult task to achieve without support due to the complex interactions among relevant factors. Directed acyclic graphs (DAG) and causal Bayesian networks (BN) offer a way to explicitly outline the underlying disease, contamination and diagnostic processes, and to further make quantitative inference on the event of interest thus serving as a tool for decision support.

**Methods:**

We prospectively collected data on children present to ED with suspected UTIs. Through knowledge elicitation workshops and one-on-one meetings, a DAG was co-developed with clinical domain experts (the Expert DAG) to describe the causal relationships among variables relevant to paediatric UTIs. The Expert DAG was combined with prospective data and further domain knowledge to inform the development of an application-oriented BN (the Applied BN), designed to support the diagnosis of UTI. We assessed the performance of the Applied BN using quantitative and qualitative methods.

**Results:**

We summarised patient background, clinical and laboratory characteristics of 431 episodes of suspected UTIs enrolled from May 2019 to November 2020. The Expert DAG was presented with a narrative description, elucidating how infection, specimen contamination and management pathways causally interact to form the complex picture of paediatric UTIs. Parameterised using prospective data and expert-elicited parameters, the Applied BN achieved an excellent and stable performance in predicting *Escherichia coli* culture results, with a mean area under the receiver operating characteristic curve of 0.86 and a mean log loss of 0.48 based on 10-fold cross-validation. The BN predictions were reviewed via a validation workshop, and we illustrate how they can be presented for decision support using three hypothetical clinical scenarios.

**Conclusion:**

Causal BNs created from both expert knowledge and data can integrate case-specific information to provide individual decision support during the diagnosis of paediatric UTIs in ED. The model aids the interpretation of culture results and the diagnosis of UTIs, promising the prospect of improved patient care and judicious use of antibiotics.

**Supplementary Information:**

The online version contains supplementary material available at 10.1186/s12874-022-01695-6.

## Introduction

Urinary tract infections (UTIs) are a common reason for children to present to hospital emergency departments (EDs) [1, 2]. Diagnoses of UTIs in children are made difficult because signs and symptoms are often non-specific and poorly sensitive, especially in those too young to communicate verbally [3, 4]. Although urine culture is considered the gold standard for the diagnosis of UTIs, urine testing may be affected by false positive and false negative results. Sample collection is challenging and urine contamination in children is frequent and can cause false positive diagnoses or obscure true positive infections, resulting in inappropriate treatment [4]. Urine testing may also be affected by false negatives due to prior use of antibiotics and low bacterial counts [3]. Management of UTIs in children requires timely decisions that balance the risks of secondary bacteraemia and sepsis if appropriate treatment is delayed, and the potential side effects of those treatments, as well as the growing public health risks from antimicrobial resistance associated with indiscriminate treatment [5].

Formulating a diagnosis relies on gathering, requesting, and synthesising information from multiple sources under time and resource constraints. Cognitive heuristics (i.e. short cuts) allow decisions to be made quickly and with little information and high uncertainty; however, these heuristics may be biased and are thought to contribute to 75% of misdiagnoses [6, 7]. The management of children with suspected UTI in the ED could benefit from decision support based on quantitative modelling. A number of predictive models have been constructed to aid the diagnosis and management of UTIs in children, with varying success. Individual biomarkers have been proposed for guiding diagnosis [8, 9], treatment and prognostication, while others propose combining routinely collected information to provide quantitative risk-based assessments [10, 11]. The lack of explanability and user engagement may be the reason many predictive models, regardless of their accuracy, fail to be successfully implemented or utilised [12–15]. Causal directed acyclic graphs (DAGs) can be used to map and describe effects centred around a causal question of interest [16], providing a potential way to address the lack of explanability.

Causal DAGs are a graphical representation of variables of interest and their relationships with each other, depicted by a series of nodes (variables) and arrows (the causal relationships between the connected variables) [17]. They assist in understanding when and how observing one variable should change our expectation of another, either because the first variable *causes* the second, the first is *caused by* the second, each is *caused by a third* variable, or because each *shares an effect* which is also observed. Arguably, causal models appeal to a type of reasoning familiar to clinicians about the unobservable (latent) pathophysiological processes which underlie disease. In contrast, rule-based decision tools simply focus only on what can be directly observed, detaching clinicians from any need to think about the underlying processes. Rule-based decision tools provide a simple heuristic for clinicians, avoiding the challenge of obtaining robust (quantitative) inference based on a DAG structure.

Bayesian network (BN) models extend DAGs by quantifying the strength and direction of the cause-effect relationships between variables using conditional probability tables (CPTs) [18, 19], providing a way to obtain formal quantitative inferences under a causal framework. When the relationships between all relevant observable and unobservable (latent) variables are organised under a causal BN framework, observed variables (data) can then be used to make probabilistic inferences about missing variables that are either unobservable and must always be inferred (e.g., latent states), or those that are potentially observable but not yet observed (e.g., future outcomes). They provide an approach for designing decision support tools by predicting unobserved variables using available data.

Causal BNs can describe a complex problem by synthesising expert opinion on the qualitative structure (i.e., the DAG), and expert opinion and/or data to parameterize it [19, 20]. Incorporating medical expertise into the building of BNs requires specialised knowledge in both the problem domain and the modelling technologies; as a result, the usefulness of BN models often increase when multidisciplinary teams work together [21, 22]. Despite the increased amount of investment (of expertise thus effort and time) required to create the models, working with medical experts to co-develop BNs compensates for data limitations that can be both systematic and significant. This improves model predictions and helps to illuminate the clinical problem at hand, increasing the likelihood that any decision support tools arising from these models will be understood, accepted and hence used in clinical care [23].

Causal BNs are now recognised as an important method for decision support in medicine especially among non-communicable diseases [21, 22]. However, evidence of implementation of BNs in healthcare settings is rare, and we believe this is partly attributable to a lack of systematic documentation from existing applications on how their BNs were developed (structure and parameterisation) from data and expert knowledge, why particular development processes were chosen, and whether those processes are repeatable [21, 24]. In this work, we propose the use of BNs for organising information in a coherent way that captures the complex relationships amongst variables relevant to the problem domain of paediatric UTIs. We describe the methodological process of building a causal BN based decision support tool for diagnosing the causative pathogens for children who present to ED for suspected UTI. We illustrate how an expert-elicited causal DAG can be translated into an applied BN model parameterised with a prospective paediatric cohort. We discuss the potential use of the applied BN model in clinical settings with the aim of guiding the diagnosis and management of UTI in children.

## Methods

This project is described in three phases to illustrate how prospective cohort data “The prospective paediatric emergency department cohort” section and an expert-elicited causal DAG “Qualitative model: the Expert DAG” section can be utilised to derive a clinical decision support BN quantifying the strength of these relationships “Quantitative model: the Applied BN”.

### The prospective paediatric emergency department cohort

Our prospective cohort enrolled children from the ED of Western Australia’s sole tertiary public children’s hospital (Perth Children’s Hospital). The study aimed to capture clinical and laboratory information about UTIs and their risk factors from paediatric ED clinicians, laboratory results, and from parents of children with a suspected UTI. A child was included if they were aged less than 13 years, presented to the ED with a suspected UTI, had urine collected for laboratory culture and susceptibility testing, were prescribed empiric antibiotics for their suspected UTI, and had informed consent provided by their legal guardian. Participants could be re-enrolled if they presented to the ED at least 14 days after their initial presentation. Ethics approval was granted by the Child and Adolescent Health Service Human Research Ethics Committee (EC00268).

Electronic and paper medical records were systematically reviewed to capture the participant’s clinical history including their demographics, reported signs and symptoms, clinical observations, laboratory results, and treatments prescribed. A standardised case report form was developed in the Research Electronic Data Capture (REDCap) system and trained research nurses reviewed and entered data in accordance with a standard operating procedure in order to accurately transcribe medical information. Parents were surveyed electronically at enrolment to identify any additional risks factors for antimicrobial resistance and 14 days after presenting to the ED to ascertain treatment outcomes. Samples were processed, analysed and reported by the local laboratory per their standard procedures. Additional file 1 provides a detailed schematic of participant enrolment and data collection.

### Qualitative model: the Expert DAG

A qualitative causal DAG was constructed based on knowledge elicited from clinical domain experts over multiple workshops and collaborative meetings. We call this the *Expert DAG*, as it details the experts’ understanding of the problem domain without the technical considerations required of a robust operational quantitative model. The experts were chosen to represent a range of health professionals involved in the diagnosis and management of children with UTIs at a tertiary hospital, and are the intended end-users of a decision support tool resulting from this work. The domain experts were from paediatric emergency medicine, microbiology and infectious diseases, general paediatrics, nephrology, epidemiology and medical laboratory science.

The elicitation rested on an initial causal framework based on preliminary insights from the prospective cohort data and mixed domain and modelling knowledge from a core team (YW, JAR, SM, TLS). Proposed relationships from this initial framework were then confirmed, corrected, or expanded after input from the broader expert group (DAF, AJC, PI, MLB, CCB, NGL, TR, AOM, PCMW). Many causal relationships between model variables were fairly intuitive and not controversial, meaning the relationships were clear (often visible) events occurring in clear temporal sequence. Therefore, elicitation of the model structure occurred with moderated discussion where a full Delphi protocol was not warranted. Additionally, discussions within a diverse expert group allowed consensus to be achieved, with specialty input only requested when needed, replicating decision-making processes in clinical care.

The outcome DAG elicited from the experts was then refined by the core team and re-presented in a written format, with each causal relationship depicted explicitly described. Further iteration was sought via written feedback and one-on-one expert and core team discussions. The resultant final Expert DAG describing the diagnosis and management of UTIs in children is described in “Expert DAG description” section.

### Quantitative model: the Applied BN

The final Expert DAG was converted into an application-oriented BN (the *Applied BN*), designed to illustrate how BN models can provide clinical decision support for the diagnosis and management of suspected UTI in children who present to the ED. Information from both the Expert DAG and the prospective cohort data were integrated to inform the selection of Applied BN variables. Conversion of the Expert DAG took into consideration: how a particular variable is relevant to the Applied BN’s purpose; how it could be matched to available data; and how it could help simplify parameterisation or computational workload. This process frequently involved simplifications by removing and merging variables, and expansions by splitting and adding variables. All changes during the conversion ensured the structure of the Applied BN was compatible with the Expert DAG, meaning all the elicited causal relationships were preserved either by explicit causal links or, where it was considered necessary, non-causal approximations.

The Applied BN was parameterised using data from the prospective cohort. In many cases, a variable’s probability conditional on its parents (predecessor node) could be estimated directly from the data. However, some of the variables in the Applied BN are latent, as they play crucial explanatory or simplification roles, and parameterisation in such cases is less straightforward. There are two kinds of latent parameters associated with latent variables: parameters that quantify the relationship of the latent variable with its parents; and parameters that quantify the relationship of the latent variable with its children (nodes extending from other nodes). In most cases, latent parameters were handled by eliciting estimated probabilities from experts and using these estimates as seeds to the expectation maximisation (EM) algorithm [25].^1^ Specifically, parameterisation surveys were created and issued to experts to elicit parameters for all and only latent variables and these parameters were used to inform the corresponding CPTs. This was not done for other (observable) variables, as sufficient data was available for such variables. In most cases, these CPTs constituted priors that were further updated by the prospective cohort data, while in other cases, the CPTs were kept fixed. In addition, one group of latent parameters were determined separately, making use of EM in the form of a clustering algorithm to “complete” the data (see Sect. 3.3 for description). Additional file 2 includes the full list of survey questions used to elicit parameters for the Applied BN, and in Additional file 6, we include all responses received for the parameterisation survey questions.

The Applied BN was evaluated from the perspective of both (numerical) accuracy and clinical usefulness. BN predictions for a selected set of target variables (e.g., pathogen-specific urine culture results) were compared with the observations of those variables captured in the prospective cohort study. The difference between the BN predictions and observations were described using two metrics based on k-fold cross-validation, namely the area under the receiver operating characteristic curve (AUROC) and the log loss [26], both intended to measure the performance characteristics of the model, though each in different ways. A sensitivity analysis was conducted on the conditional probability parameters with a high degree of uncertainty, using variance-based sensitivity analysis (VBSA) [27, 28]. VBSA allows the distribution of several input parameters to be investigated simultaneously to help understand how changes influence the BN target predictions in the CPTs. The clinical experts evaluated the clinical usefulness of the BN via a validation workshop where relationships and concepts were checked and refined. Three scenarios were simulated to demonstrate how the Applied BN might be used for clinical decision support for a child presenting to the ED with a suspected UTI.

## Results

### Prospective paediatric cohort

From May 2019 to November 2020, 391 children were enrolled in the prospective cohort study. This accounted for 431 UTI episodes, where the mean age at presentation was 3.9 years old (Interquartile Range, IQR, 0.7—6.2) and 316 (73%) were girls. A prior history of UTI or urinary tract pathology (e.g., neuropathic bladder, phimosis, renal agenesis, dysplasia) were reported in 197 (46%) of participants according to their medical notes or reported by their parent in the study survey. Commonly reported symptoms on ED presentation included parent-reported fever (269, 62%), nausea and/or vomiting (169, 39%), poor oral intake (161, 37%), abdominal pain (144, 33%), and pain or discomfort referrable to the urinary tract (148, 34%). Symptoms varied significantly between those < 2 years old and those ≥ 2 years old (Table 1). Children were prescribed antibiotics during their episode of care as per the inclusion criteria, where broad spectrum^2^ antibiotics were prescribed in 32% of children. Among the 431 urine samples collected in the ED, 219 (51%) reported pure growth, 150 (35%) reported no growth and 7 (2%) reported mixed growth, while urine culture data was unavailable for 56 episodes (13%). *Escherichia coli* (*E.coli*) was the most common bacteria reported accounting for 204 (47%) of total episodes and 90% of positive urine samples (204/226). Other Gram negative organisms (e.g. *Proteus mirabilis, Enterobacter cloacae, Pseudomonas aeruginosa*) and Gram positive organisms (e.g. *Staphylococcus aureus, Enterococcus faecalis*) were isolated in 4% and 3% of total episodes, attributing to 7% (16/226) and 6% (13/226) of positive samples, respectively. Antibiotic use prior to ED presentation was reported in 61 (14%) of episodes and was negatively associated with urine culture (Table 1).

**Table 1:** Prospective cohort study summary statistics

***Demographics and clinical history***		
**Subgroup by age group**	** < 2yo 179 (41.5% of total 431 episodes)**	** > = 2yo 252 (58.5% of total 431 episodes)**
Female	101 (56%)	215 (85%)
Prior urinary tract pathology (including previous UTI)	55 (31%)	142 (56%)
On antibiotics at ED presentation^2^ Broad Narrow	6 (3%) 15 (8%)	14 (6%) 26 (10%)
***Clinical symptoms recorded***		
Pain or discomfort referrable to the urinary tract (e.g., dysuria, genital pain)	11 (6%)	137 (54%)
Parent reported fever	137 (77%)	132 (52%)
Temperature > 38 °C	43 (24%)	64 (25%)
Abdominal pain	5 (3%)	139 (55%)
Foul smelling urine	34 (19%)	27 (11%)
Haematuria	6 (3%)	17 (7%)
Irritable	72 (37%)	19 (8%)
Lethargy	52 (29%)	51 (20%)
Nausea/vomiting	76 (42%)	93 (37%)
Poor oral intake	85 (47%)	76 (30%)
Diarrhoea	25 (14%)	12 (5%)
Respiratory symptoms	46 (26%)	43 (17%)
***ED Investigations and management recorded***		
C-reactive protein ≥ 15 mg/L Investigation not done	49 (27%) 108 (60%)	29 (12%) 206 (82%)
Leucocyte count ≥ 10 × 10^9/L Investigation not done	57 (32%) 109 (61%)	30 (12%) 209 (83%)
Neutrophil count ≥ 8 × 10^9/L Investigation not done	26 (15%) 109 (61%)	28 (11%) 209 (83%)
Broad spectrum^2^ antibiotic empirically prescribed	57 (32%)	82 (33%)
Patients discharged after ED consult	108 (60%)	217 (86%)
***Urine analysis***		
Method of urine specimen collection Clean catch Catheter Suprapubic aspirate	63 (35%) 70 (39%) 2 (1%)	112 (44%) 16 (6%) 0 (0%)
Bacteria seen on microscopy	110 (66%)	94 (37%)
> 100 leucocytes per high power field	107 (60%)	145 (57%)
Moderate epithelial cells on microscopy	21 (12%)	32 (13%)
Leucocyte esterase (3 +) on urine dipstick	51 (28%)	100 (40%)
Nitrites detected on urine dipstick	64 (36%)	76 (30%)
***Urine culture***		
No growth	47 (26%)	103 (41%)
*E.coli*	97 (54%)	107 (42%)
Gram-negative bacteria (other than *E.coli*)	6 (3%)	10 (4%)
Gram-positive bacteria	5 (3%)	8 (3%)
**Subgroup by antibiotics use prior to ED**	**On antibiotic 61 (14%, *n* = 431)**	**Not on antibiotic 342 (79%, *n* = 431)**
No growth	35 (57%, *n* = 61)	106 (31%, *n* = 342)
*E.coli*	13 (21%, *n* = 61)	177 (52%, *n* = 342)
Gram-negative bacteria (other than *E.coli*)	4 (7%, *n* = 61)	11 (3%, *n* = 342)
Gram-positive bacteria	2 (3%, *n* = 61)	11 (3%, *n* = 342)

Unless stated otherwise, all percentages were calculated using positively reported observations within each age group (i.e., as a percentage of the 179 cases for < 2yo, and 252 cases for >  = 2yo). Of note, when a variable (e.g., abdominal pain) was not reported, it’s likely that the child reported no pain (confirmed negative observation) or the data was missing (e.g., not queried or recorded by the treating doctors)

### Expert DAG description

**Fig. 1:**
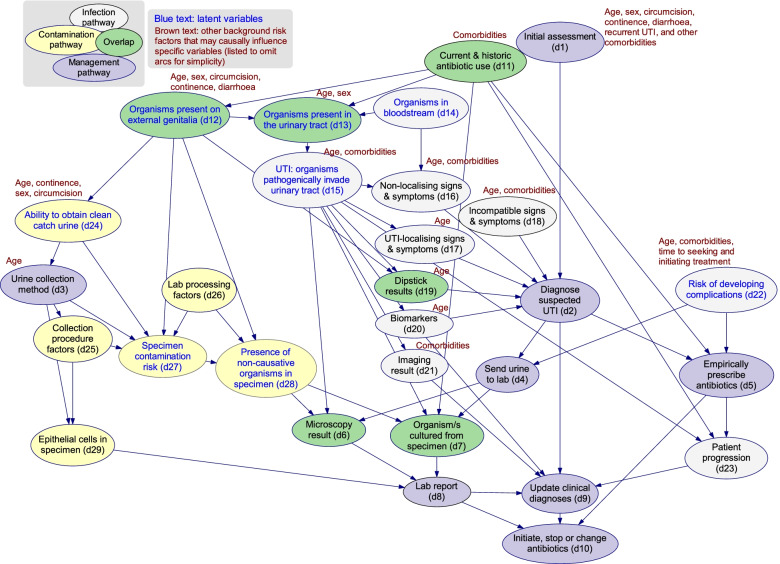
The Expert DAG v11.1. The expert-elicited causal directed acyclic graph describing the relationships between infection (white), specimen contamination (yellow) and UTI management (purple) in children, in particular, variables that fell into more than one pathways were indicated in green. Note: Numbers within the model nodes correspond with the narrative description. A detailed variable dictionary is provided with the supplementary material: Additional file 3. The source model file for the Expert DAG can be accessed via the Open Science Framework [30]

The Expert DAG comprising 29 variables represents a mechanistic causal model of UTI infection, diagnosis and management of children presenting to an ED (Fig. 1). The model can be divided into the infection, contamination, and management pathways. In Additional file 3, we provide a detailed variable dictionary for the Expert DAG describing the meaning of each variable, the interactions modelled, and the causal mechanisms involved.

#### The Infection Pathway

The infection pathway describes predisposing background factors and the pathophysiology of infection, and how a UTI gives rise to signs, symptoms and laboratory evidence. For a UTI to occur, organisms must be present in the urinary tract (d13), usually from ascension of organisms from the external genitalia (d12) or, on rare occasions, from haematogenous seeding of the upper urinary tract with organisms from the bloodstream (d14) which then infect the urinary tract (d15) [31]. Infection here is a ‘latent’ event, meaning that although it may be inferred from evidence with varying confidence, it generally cannot be directly observed; importantly we separate the *existence* of a *UTI* (infection pathway, d15), from the *diagnosis* of a *suspected UTI* (management pathway, d2) based on the presence or absence of various signs, symptoms, dipstick test and laboratory results. Age and UTI-relevant comorbidities (such as structural or functional abnormalities of the urinary tract) influence the probability of a UTI in a given child, due to their predisposing effect [32]. Infection of the urinary tract typically provokes an inflammatory response which may manifest as UTI-localising signs and symptoms (d17) caused by inflammation of the urinary tract, and/or non-localising signs and symptoms (d16) caused by systemic inflammation. In children, especially those too young to communicate verbally, UTI-localising symptoms may be difficult to ascertain, forcing clinicians to assess observable signs and symptoms that are non-specific and non-localising such as fever and irritability, and which are shared with other conditions [33]. Where incompatible signs and symptoms (d18) are present—those not typically associated with a UTI (e.g., respiratory symptoms), the diagnosis is dependent on the probability of alternative diagnoses that may provide a better explanation for the child’s presentation.

#### The contamination pathway

The practical definition of urinary contamination varies widely across the literature and in practice [34, 35]. Contamination and infection are often considered mutually exclusive, but in reality organisms cultured from urine samples may be pathogens, contaminants, or both. In our model, contamination is treated as a latent event, and describes the presence of non-causative organisms in a urine specimen (d28). Contamination usually occurs at the time of collection when organisms present superficially on the external genital area (d12) become mixed with the ‘clean’ urine sample from the bladder (in this context, ‘clean’ means the specimen is free from contaminants, not that it is free of organisms). A child’s age, sex, and for boys, circumcision status, can directly influence both the density of any organisms present on the external genitalia (d12) and the ability to produce a clean urine sample (d24). Incontinence and/or diarrhoea may increase the density of organisms present on the external genitalia (d12), increasing the risk of specimen contamination (d27), and possibly also the risk of infection of the urinary tract (d15) via the ascending route (d12).

The probability of contamination is strongly influenced by the urine collection method (d3). Within the model, the latent concept of specimen contamination risk (d27) represents all factors contributing to contamination which, if true, increases the probability of the presence of non-causative organism in the specimen (d28). Laboratory processing factors (d26) representing any process that may introduce (rare in most laboratories) or concentrate non-causative organisms in the specimen (d28) from when the urine arrives in the laboratory to its final reporting. This may include delays in sample processing or refrigeration and improper aseptic technique.

#### The management pathway

The existence of a UTI cannot be known with absolute certainty, and a clinician’s belief (or judgement) about its presence or absence may vary over time, perhaps related to evolving evidence. It may be suspected on the clinician’s initial assessment (d1) based on the child’s history and background risk factors. As more evidence is gained via the elicitation of symptoms and signs and from investigations, a working or provisional diagnosis of UTI is made (d2) – thus, the suspicion based on the initial assessment (d1) is updated. A urine specimen may be sent to the laboratory (d4) and if the suspicion of UTI is sufficiently high, empiric antibiotics may be prescribed (d5) even before the urine testing results are known. Management decisions are also influenced by whether the clinician believes that there is a high risk of the patient having or developing complications (d22). In the model, this is represented as a latent concept that describes the risk of progressing to severe complications. This risk is largely driven by a child’s age, the time delay to seeking and/or initiating treatment, and the presence of comorbidities such as abnormalities of the urinary tract or immune system.

Interpretation of the presence, type and density of growth cultured from a urine specimen (d7) is difficult, as this is where the contamination and infection pathways converge. Information regarding these pathways is not normally available to the laboratory scientist deciding how to report the results (d8) of the urine test. Thus, if an organism is isolated with evidence of an inflammatory response (e.g. pyuria) on microscopic analysis (d6), the probability that the cultured organism is causative is high and therefore it is reported as significant in the laboratory report (d8) and antimicrobial susceptibility results are also reported. In contrast, the isolation of multiple organisms is typically reported as a ‘mixed growth’, precluding either the confirmation or exclusion of a UTI.

A final updated clinical diagnosis (d9) is made when outstanding evidence or other information is available. The existence of a UTI directly influences the urine laboratory report (d8), any biomarker (d20) and imaging results (d21), as well as the subsequent clinical progress of the child (d23) with or without antibiotic treatment. A clinician uses these observations to further update their belief about the probability that the patient has a UTI, together with any antimicrobial susceptibility data from the laboratory report (d8) to decide whether to initiate, stop or change the antibiotic prescription (d10).

### Applied BN for decision support

**Fig. 2:**
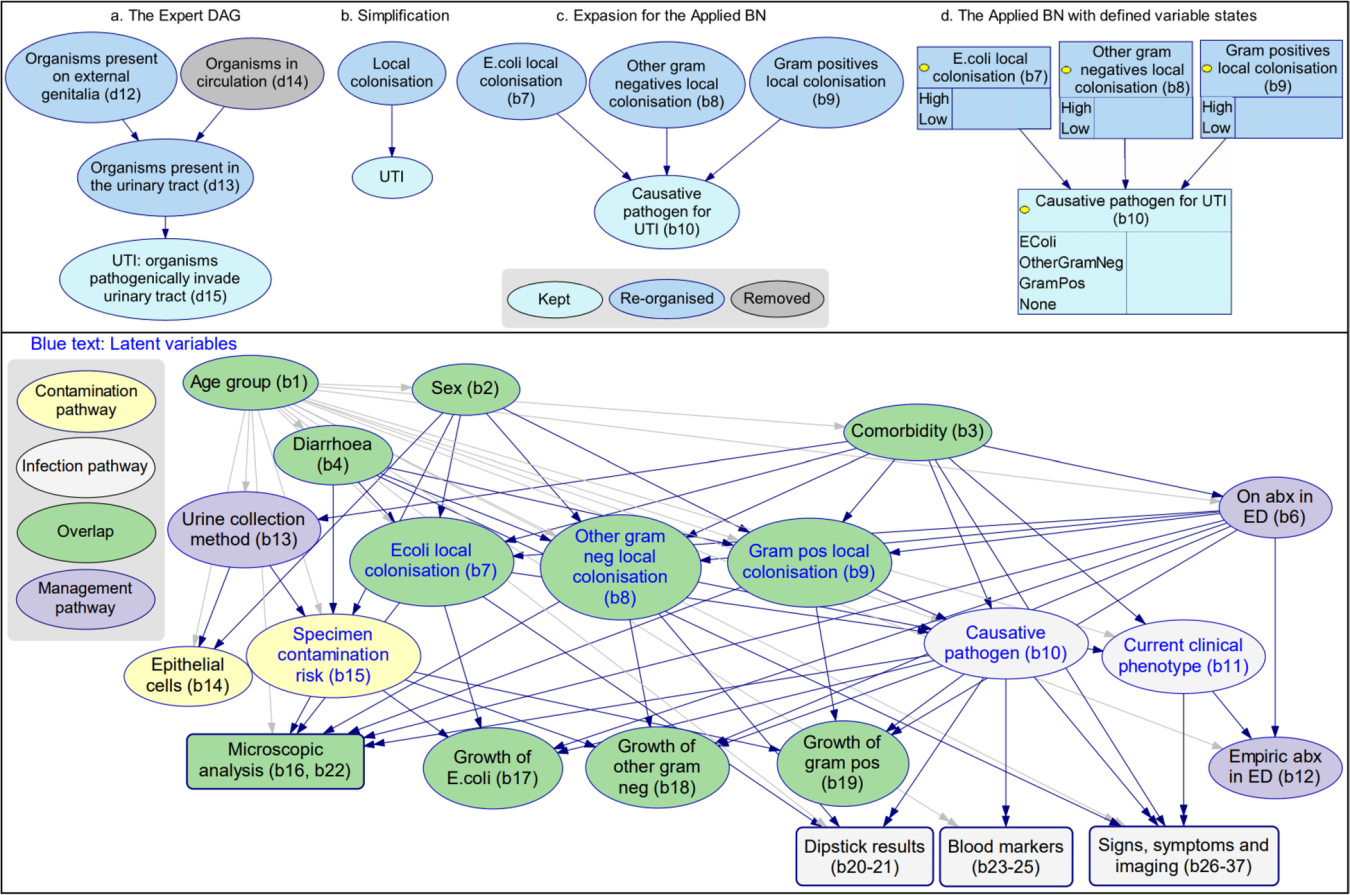
Top: An example of converting from the Expert DAG v11.1 to the Applied BN v2.2. Bottom: The high-level Applied BN structure. Additional file 4 includes a full list of differences between the two models. Additional file 5 presents the detailed structure of the Applied BN, in particular the local structure of submodels microscopic analysis, dipstick results, blood markers, and signs and symptoms (round box in the bottom panel), as well as the BN variable dictionary. The source model file for the Applied BN can be accessed via Open Science Framework [30]

The Applied BN represents a demonstrative decision support tool using the Expert DAG that aims to help determine if a child truly has a UTI and if so, the likely causative pathogen. To develop this BN, variables in the Expert DAG were mapped to available data from the prospective cohort. Conversion of the Expert DAG into the Applied BN required simplification and expansion, whilst ensuring compatibility and preservation of the causal knowledge. Illustrative steps are summarised along the top of Fig. 2. In this example, a fragment of the Expert DAG is selected (step a) that describes the presence or colonisation of bacterial pathogens on the external genitalia and in the urinary tract using two variables (d12 and d13), with an arc between them indicating that pathogens may spread from the genitalia to the urinary tract. In addition, there is depicted another possible (albeit uncommon) pathway for a pathogen to reach the urinary tract haematogenously via the bloodstream (d14). For simplicity, d14 was removed, and since this left only one explicit pathway, d12 and d13 were combined into a single variable that broadly describes local colonisation (step b). The local colonisation variable was then expanded (step c) into three nodes (b7-9) to describe local colonisation for three specific pathogen groups which are of key interest and that not only affect the probability of developing UTI, but may also constitute the causative pathogen if UTI is present (b10). Variable states were then selected (step d), typically to match the data where possible. However, in the case of latent states, this was not possible and the goal instead was to represent key divisions within each variable while minimising the demand on the latent parameterisation process. Here, each local colonisation variable is latent and has been assigned two states (High and Low), with the causative pathogen variable being assigned four states (one state for each possible causative pathogen plus a state for no pathogen/no UTI). The causative pathogen was assumed to be singular and mutually exclusive, i.e., assuming no co-infection of the urinary tract by two or more pathogens. Additional file 4 includes a full list of differences between the two models.

The Applied BN (Fig. 2, bottom panel) comprises 36 nodes including 6 latent nodes, which can all be mapped to variables in the Expert DAG (see Additional file 4). Expert survey responses were collated (Additional file 6) to inform the CPT priors for the BN, which were further updated by training based on the prospective cohort data (as described in “Quantitative model: the Applied BN”) section. Of note, the node ‘current clinical phenotype’ was introduced into the Applied BN as a summary node of patient presentation phenotypes after feedback from the expert validation workshop. This node is latent but was treated uniquely to provide a definition of current clinical phenotype that is independent of other latent factors in the model. In particular, a separate clustering was performed (using the EM algorithm) on the signs and symptoms, resulting in a grouping into three types, simply called “Type 1”, “Type 2” and “Type 3”, “Type 1” being systemic signs and symptoms predominant but mild urinary tract localising symptoms, “Type 2” being urinary tract localising symptoms predominant, and “Type 3” being abdominal pain predominant with minimal other symptoms. The clustering model was then used to determine each patient’s most probable clinical phenotype, and this information was added to the prospective cohort data in the form of an additional column and subsequently treated like an observed variable.

**Fig. 3:**
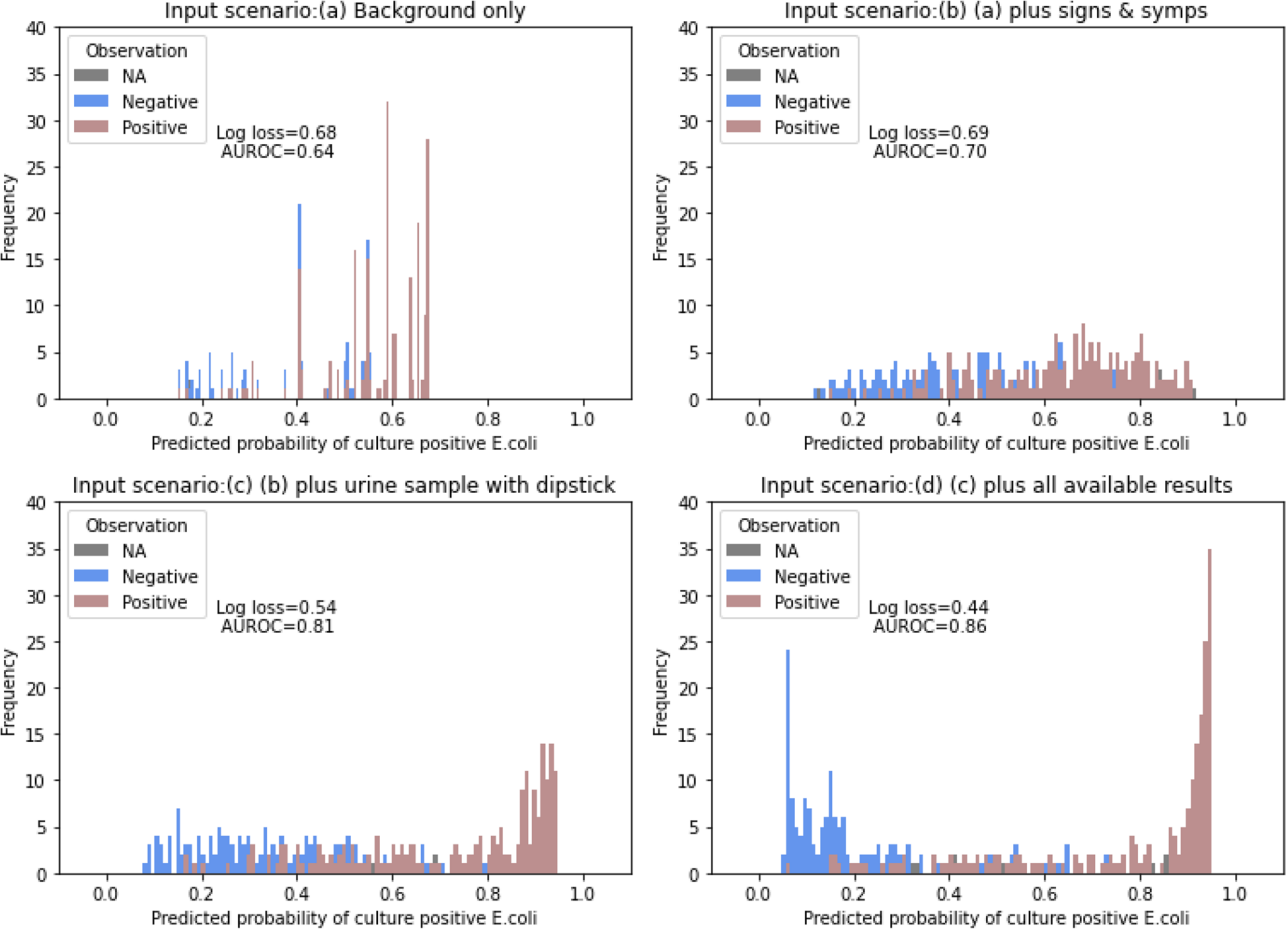
Applied BN v2.2 performance as compared with observations, with Log Loss and AUROC across four scenarios. Each panel presented the distribution of the Applied BN predicted probabilities of isolating *E.coli* from urine sample given available patient’s information under the specified scenario. The predicted probabilities were compared with the reported culture result of each patient, where brown, blue and grey indicated *E.coli* was isolated, not isolated and no data, respectively. Scenario (**a**): age, sex, history of UTI, urinary tract comorbidities. Scenario (**b**): scenario (**a**) + reported diarrhoea, urine tract pain or discomfort, abdominal pain, haematuria, foul smelling urine, respiratory symptoms, parent reported fever, temperature, irritability, lethargy, nausea/vomiting, poor oral intake. Scenario (**c**): scenario (**b**) + urine collection methods, urine dipstick results (leucocyte esterase & nitrite). Scenario (**d**): scenario (**c**) + urine microscopy (leucocytes, bacteria, epithelial cells), leucocyte and neutrophil count (from full blood count), C-reactive protein level and ultrasound result

It is important to reiterate, by UTI, we mean the *existence* of UTI, which reflects the state of the world where a child’s urinary tract is infected by a pathogenic organism, and is only imprecisely defined. As a result, operational definitions of UTI and its causative pathogen vary across studies, and the definition is often incomplete (missing cases that we want to classify as UTIs). Evidence for UTI is indirect and comes from factors like cultures results and expert judgements, which is the way we approached it with our BN, leaving UTI as a latent variable and defined by its relationship with these other factors. The primary BN output is the causative pathogen for UTI (b10). Results from a 10-fold cross-validation^3^ show that the model predicts 68.0% of the presenting episodes in our cohort were UTIs, with IQR 67.2–68.9%. Specifically, this includes 40.2% *E.coli* UTI (IQR 39.3–40.8%), 11.6% other Gram negative UTI (IQR 11.6–11.9%), and 16.3% Gram positive UTI (IQR 14.9–18.0%). Figure 3 presents the Applied BN predictions for *E.coli* culture for every presenting episode, and compares these against their final laboratory results. The graphs represent four scenarios (a-d), each providing more information to the model than its preceding scenario. Namely, (a) provides the model with information on *basic demographics (age and sex) and clinical history (history of urinary tract pathology)*, (b) provides *(a) plus reported signs and symptoms*, (c) provides *(b) plus urine collection method and dipstick results*, and (d) provides *(c) plus all other available results (including urine microscopy and other clinical investigations).* The evaluation results show that the evaluation metrics (log loss and AUROC) improve as more evidence is available for a given child, especially if that evidence is sensitive and/or specific for UTI.

Two sets of parameters turned out to be very important in driving the primary target of the Applied BN (i.e., Causative pathogen, b10), namely, the probability of UTI in the prospective cohort (i.e., one minus the probability that Causative pathogen is none) and the pathogenicity (i.e., likelihood of causing disease and worsening illness) for each organism. Understanding the proportion of UTI and the pathogenicity of different organism groups is key as they determine how often a child would acquire UTI given local colonisation of an organism that is potentially pathogenic, which organism is more likely to be the causative pathogen when two or more organism groups co-colonise, and how likely the child would manifest as a more severe clinical case. These parameters were challenging to estimate as they were completely latent, hence we relied on expert opinion collected via a parameter survey as described earlier (Sect. 2.3). For the first of these parameters, the survey responses gave a mean estimate of 68% UTI among the study cohort (IQR 59–81%). Table 2 presents the survey outcomes for the second set of parameters on pathogenicity for each organism. We defined the pathogenicity of an organism as the propensity of the organism to cause UTI when an otherwise healthy child is colonised by that organism on the perineum or external genitalia. The survey elicited the pathogenicity of other Gram negative and Gram positive organisms as a numerical ratio relative to *E.coli*, on average, the responses suggest that *E.coli* and gram positive bacteria are very similar regarding their pathogenicity (1 and 0.98 respectively), and the other Gram negative bacteria is the most pathogenic (scored 1.35). Unlike other responses in the survey, the elicited responses for pathogenicity varied widely among experts. In Additional file 6, we provide a summary of responses to all survey questions.

**Table 2:** Expert survey outcome results of organism pathogenicity

**Pathogenicity**	**Average**	**Standard Deviation**	Expert 1	Expert 2	Expert 3	Expert 4	Expert 5	Expert 6	Expert 7	Expert 8
*E.coli*	1	0	1	1	1	1	1	1	1	1
*Other Gram negatives*	1.35	0.53	1.5	2	0.8	1	0.75	1.75	1	2
*Gram positives (e.g., Enterococcus)*	0.98	0.91	0.5	0.2	0.5	1	0.375	2.75	0.5	2

**Fig. 4:**
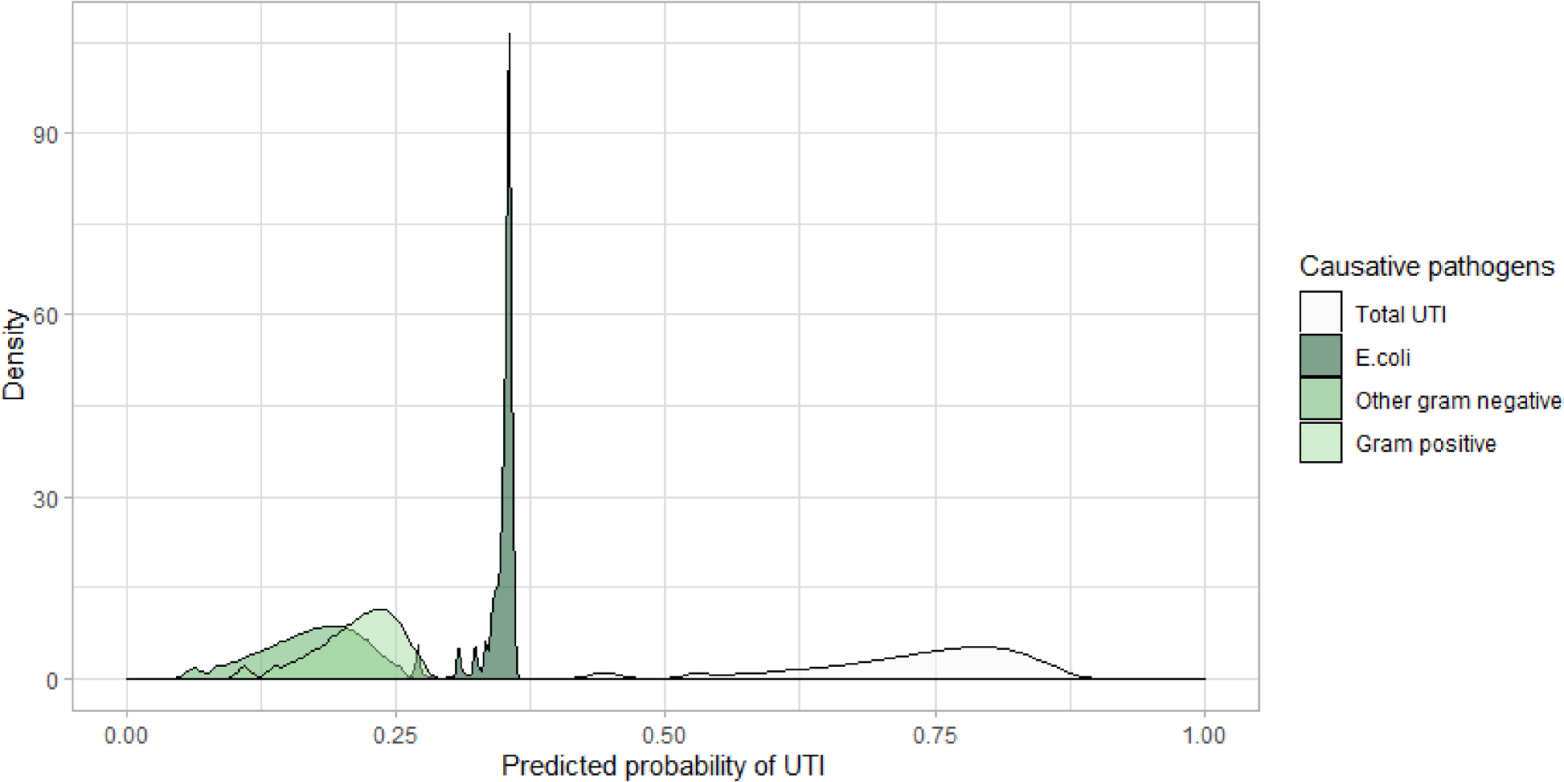
Sensitivity analysis on causative pathogens as the pathogenicity of different organism changes by ± 20%

Given the high level of variation in these survey outcomes, we therefore conducted sensitivity analyses by varying the prior CPT parameters for b10 by ± 20%. In response, as shown in Fig. 4, the predicted probability of UTI in our cohort of suspected UTIs ranged from 44 to 87%. *E.coli* is always predicted to be the most likely causative pathogen among the UTIs (39–64%), the relative attribution of other Gram negatives and Gram positives as the causative pathogen among the UTIs is sensitive to their pathogenicity, ranging from 12–29% and 22–32%, respectively.

**Fig. 5:**
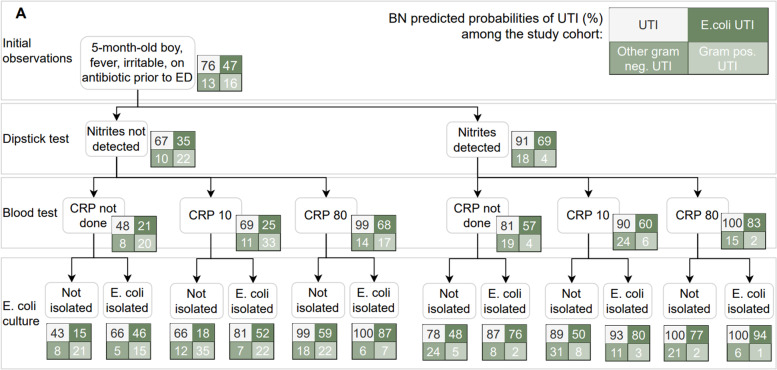
Predictions from the Applied BN under the clinical scenario **A**

**Fig. 6:**
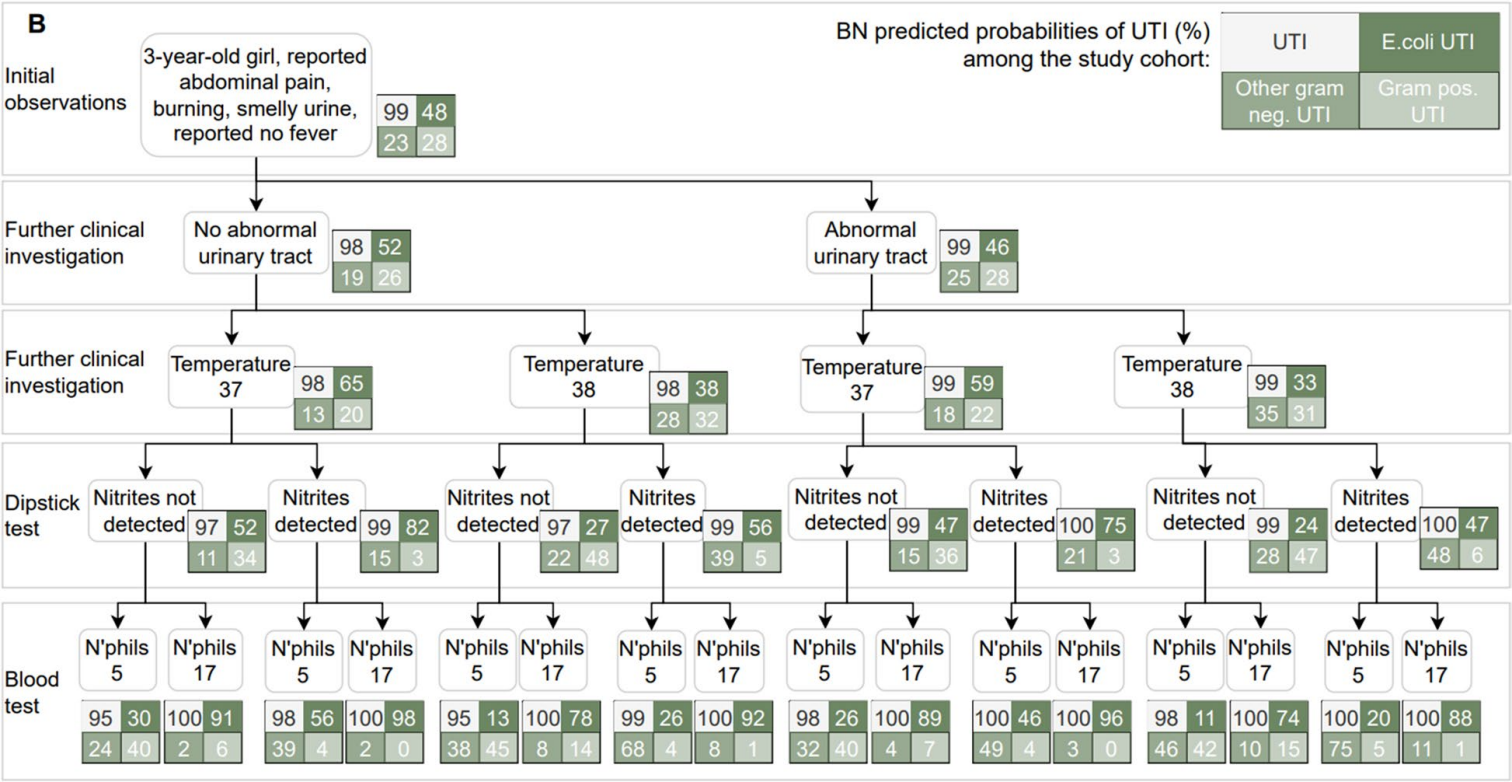
Predictions from the Applied BN under the clinical scenario **B**

**Fig. 7:**
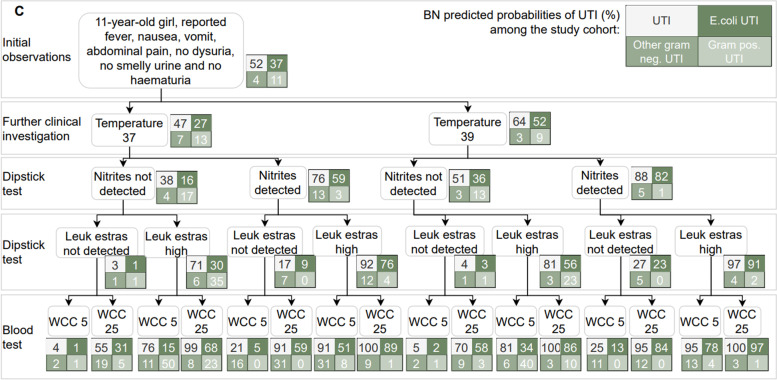
Predictions from the Applied BN under the clinical scenario **C**

Figures 5, 6 and 7 present three hypothetical clinical scenarios to illustrate how the Applied BN may be used for point of care decision support in the management of children with a suspected UTI. Predictions for each of the scenarios is shown branching conditional on various potential information and test results as they may become available over time. The scenario in Fig. 5 presents an infant who is unable to communicate any localising symptoms. As information from the dipstick test, blood test and culture result become available, the BN’s predictions for the presence of UTI (and, if present, the associated causative pathogen) are updated accordingly. For example, when evidence from a dipstick result and C-reactive protein (CRP) analysis are strongly indicative of UTI (i.e., “Nitrites detected” and “CRP 80”), a negative culture won’t exclude a UTI. Figure 6 presents a scenario in which UTI is always highly probable. Here, the presence or absence of comorbidities, temperature, dipstick nitrites and blood neutrophil levels only influence which causative pathogen is most likely. Finally, Fig. 7 describes a child with no obvious localising symptoms, where a combination of test results can both rule in or rule out a UTI, as well as affect conclusions about the most likely causative pathogen.

## Discussion

Diagnosis and management of UTIs in children can be challenging due to the variability of clinical presentations and difficulties in obtaining a urine sample free from contamination. By mapping the causal pathways involved in this process through the development of an expert knowledge-derived DAG (the *Expert DAG*, Fig. 1 & Additional file 3), we have highlighted how the convergence of the causal pathways through sample collection and clinical diagnosis are key in creating this diagnostic challenge. Further to this, we have described which information may be available at different stages of the diagnostic and management process, and which additional evidence may be required to better understand the causal process. With the data collected from 431 episodes of suspected UTI in children, we converted the Expert DAG into a causal Applied Bayesian network model (the *Applied BN,* Fig. 2 & Additional file 5) to assess the probability of UTI (and if so the causative pathogen) among children with a suspected UTI. The Applied BN achieved an excellent and stable performance in predicting *E. coli* culture results, with a mean AUROC of 0.86 and a mean log loss of 0.48 based on 10-fold cross-validation. We illustrated how the Applied BN could be implemented in practice as a clinical decision support tool using three hypothetical clinical scenarios.

### The need for a better understanding of epidemiology and diagnosis of UTI

UTI epidemiology is primarily described based on urine culture results which are influenced by three causal pathways; (i) specimen contamination, where bacteria are introduced and not causative of the infection, (ii) clinical management, where empiric antibiotic exposure may suppress the bacteria causing an infection and/or specimen contamination, and (iii) the pathogenic causative organism of interest. Among the 431 episodes of suspected UTIs enrolled through the prospective cohort, after excluding 55 missing culture results, 60% specified growth of a bacterial organism, of which 90, 7 and 6% were *E. coli*, other Gram negative bacteria and Gram positive bacteria, respectively. After the cohort data was used to train the Applied BN and thus interpreted under a causal framework, 69% of the study cohort were predicted to be UTIs, of which 57, 16 and 27% were predicted to have been caused by *E. coli*, other Gram negatives, and Gram positive bacteria, respectively. The difference in the predicted distribution of causative pathogens by the causal model and crude microbiology data, which does not account for contamination and the effect of prior treatment, could have implications for antibiotic guidelines and urine culture reporting protocols.

More explicitly, the observed proportion of *E.coli* culture (54% of the overall prospective cohort) does not include all and only cases of UTI. The Applied BN suggests that: (i) specimen contamination results in 26% of urine culture isolates of *E.coli* being predicted to be non-UTIs and non-*E.coli* UTIs (i.e., false positives); and (ii) 84% of predicted *E.coli* UTIs reported growth of *E.coli*, implying a 16% false negative rate with a predicted 73% of prior antibiotic use. This concept is further described in the illustrative scenario of Fig. 5, where for an irritable infant boy with fever and antibiotic use prior to ED, having no nitrites detected on urinary dipstick and with no CRP test performed, and where *E. coli* was isolated from the urine sample, the Applied BN predicts a 46% probability of this representing a *E. coli* UTI. In contrast, for the same child with nitrites detected on their urinary dipstick and with a CRP of 80 mg/L, the Applied BN predicts a 77% chance of a *E. coli* UTI, even if *E. coli* is not isolated from the urine sample.

When making decisions, clinicians are required to balance the risks associated with treatment based on a positive urine culture result that may not represent a UTI, against the risk of complications if UTIs are not adequately treated, particularly in neonates and young infants. By organising observable information within a causal DAG, we can highlight potential mediators, confounders and sources of selection bias and measurement errors [37]. The prospective cohort study has mapped out the variation in the clinical picture of children investigated for a suspected UTI. Reported symptoms and urine analysis results differed greatly with age (Table 1), which likely represents an amalgam of children with and without UTI, and further highlights the need for decision support tools to distinguish between these groups. Mapping observable variables of the prospective cohort study cohort to the variables described in the Expert DAG, coupled with simplification and expansion, has enabled a quantitative model to be developed into a decision support tool (the Applied BN). Interpreting the observations available to clinicians under this causal framework may offer a clearer understanding of the clinical picture and provide robust assessments of the likelihood of UTI.

Organism-specific pathogenicity needs to be better understood to improve the diagnosis of the causative pathogen for each UTI. Based on our experts’ survey responses (Table 2), we assumed that non-*E. coli* Gram negative bacteria have the greatest pathogenicity in the current model, while *E.coli* and Gram positive pathogens have similar and lower pathogenicity. These assumptions can have implications for the model predictions. An example can be found in the scenario of Fig. 6, where, for a 3-year-old girl with reported abdominal pain, smelly urine, burning, no parent reported fever but a recorded temperature of 38 degrees, nitrites not detected on urinary dipstick and blood neutrophil count of 5 × 10^9^/L, the Applied BN predicts other Gram negative bacteria as the most likely causative pathogen, regardless of whether the child has any history of urinary tract pathology. However, the survey outcomes indicated a high level of variation regarding the relative expert-derived pathogenicity of different organism groups. This was especially relevant for Gram positive organisms where growth is often attributed to contamination and the ability of some organisms to cause UTI may be disputed. As a result, the Applied BN only demonstrates there is a potential to differentiate causative pathogens for UTI like non-*E.coli* Gram negative and Gram positive bacteria, based on assumed pathogenicity and current data. While it’s not yet ready to be used for differentiating pathogens, it does suggest a way forward in understanding the organism-specific pathogenicity.

### Learnings from the modelling process

The Expert DAG demonstrated that specimen contamination risk, propensity to develop complications and an organism invading the urinary tract system were the key latent concepts that concerned clinical teams. Importantly, superficial colonisation of the perineum/ genitalia lies on the causal pathways mediating both invasion of the urinary tract system and specimen contamination which converge at urine culture, the only point at which either of the two pathways is typically observed. We therefore chose to model these variables explicitly in the Applied BN as pathogen-specific ‘local colonisation’ (b7-b9), ‘causative pathogen’ for UTI (b10) and ‘specimen contamination risk’ (b13), despite the challenges of parameterising these latent nodes. We addressed this challenge by designing survey questions to elicit estimates of relevant parameters from the domain experts. In some cases, even those expert elicited responses were inconclusive (namely, the organism-specific pathogenicity) and in those cases we conducted sensitivity analyses to ensure the implications and limitations of the uncertain parameters were recognised (as discussed for pathogenicity in the previous section).

Where possible, the Expert DAG was causal and comprehensive of the problem domain rather than constrained by variable observability or data availability. This allowed it to be used as an accurate representation of expert knowledge, enabling the use, adaptation and extension by the core research team and external researcher. The Expert DAG constitutes the knowledge base for creating the Applied BN, and once the BN is created, the Expert DAG is no longer involved – for example, only the Applied BN would be used as part of any decision support tool. In the ideal case, when expert understanding evolves, the DAG should be updated to reflect this new understanding, which would in turn drive future updates to the BN. By documenting the detailed steps of the conversion from the Expert DAG to the Applied BN (Additional file 4), we established a methodological framework that can be generalised beyond the UTI problem domain. Decisions to keep, remove or add variables in the applied model should be driven by a well-defined modelling purpose, matched to the availability and quality of data, and technical efficiency (such as reducing the number of latent nodes, or reducing the complexity of the variable relationships).

Like most complex modelling work, our variable selection, structure development, parameterisation and evaluation processes were iterative. The communication between modellers and the domain experts played an important role in this project, which required both parties to make efforts to understand each other’s expertise and language. Medical education focuses on the pathophysiology of disease, where factor ‘X’ predisposes to outcome ‘Y’. However, in practice, clinicians are more experienced in using rule-based flow charts and decision trees to aid in management, which depict ‘if [specific signs and symptoms], then perform [this test]; if [this result] then commence [this treatment]’. The creation of an expert-derived DAG required clinicians not only to revisit the concepts of the causal effects of each variable and their direct influence on another, but also to depart from the concept of a graph reflecting a sequence of steps or yes/no questions to observations, and instead that one may have the real outcome of interest (e.g. the *existence* of UTI) existing as a latent node in the body of the model, influencing the observable nodes that appear below it. Similarly, the concept of a latent node was challenging, given that clinicians typically work on the premise that they have the correct (i.e., ‘true’) diagnosis that informs their treatment decisions. While clinicians are certainly familiar with the related concepts of false positives and negatives, the extension to latent nodes was not straightforward and required more guidance from the core research team. The creation of a DAG highlights the fact that some important variables will always remain unobserved and therefore uncertain; although evidence may be accumulated to increase certainty about the presence or absence of infection, in reality infection can only ever be inferred and never directly observed. Becoming comfortable with these concepts enabled the experts to create the elicited DAG and understand its utility in clinical practice in the form of a BN.

### Study limitations and future research

The prospective study cohort aimed to describe participants < 13 years of age who presented into the ED and were prescribed antibiotics for a suspected UTI. With these criteria the data used to develop the models and resultant models likely represents patients that have more severe and complex disease and a greater risk of hospitalisation and antimicrobial resistance than those presenting for a UTI within the community. In other words, selection bias may be generated at the time patients were screened for eligibility and recruited for data collection, limiting the use of the Applied BN to the same cohort. Microbiology data obtained as part of the prospective study cohort was limited. The distribution of pathogens was likely representative, however, there were a small number of samples that isolated non-*E. coli* Gram negative bacteria and Gram positive bacteria. This required a broad pathogen grouping which may have included bacteria with greatly differing uropathogenic characteristics, as a result, only a limited understanding of how clinical and laboratory variables can help differentiate causative pathogens was developed. Further to this, a greater understanding of colonisation, infection and bacteria specific pathogenicity in the urinary tract is required to further the development of this model, yet much of this information is debated widely in the scientific community [38]. The Applied BN briefly describes the empiric antibiotic prescribing patterns within the ED where 62 and 38% of described antibiotics prescriptions were narrow and broad spectrum, respectively. It is intended this model will be expanded with additional information on antimicrobial susceptibility profiles to evaluate the appropriateness of empiric antibiotic prescriptions for a range of causative pathogens.

With a richer dataset, our models could benefit from further development that could provide predictions for a broader scope, for example, incorporating how decisions were made on collecting urines and conducting blood tests, as well as potential other diagnoses other than UTI. We provide this model in its current updatable form for further parameterisation, validation, and extension by external and future researchers. The model can be adapted across a range of laboratories, hospitals and patient populations, and we anticipate this framework will aid the interpretation of culture results, the diagnosis of UTIs, and choice of antibiotic prescription, and can be incorporated into routine clinical pathways with the overall goal of improving patient outcomes and reducing inappropriate antibiotic use in children. To our knowledge this is the first causal BN for UTIs in children; we believe it serves as an exemplar for the creation and use of causal model-based decision support tools across a broad range of infectious disease problems.

## Supplementary Information

**Additional file 1. **Schematic of participant enrolment and data collection.

**Additional file 2. **Parameterisation survey questions.

**Additional file 3. **The Expert DAG and variable dictionary.

**Additional file 4. **List of changes when converting the Expert DAG to the Applied BN.

**Additional file 5. **Full structure of the Applied BN and the BN dictionary.

**Additional file 6. **Parameterisation survey responses.

## Data Availability

All source models and associated dictionaries are accessible as additional files to the manuscript, and via our Open Science Framework page, https://osf.io/8taqy/.

## Abbreviations

UTI: Urinary tract infection
ED: Emergency department
DAG: directed acyclic graphs
BN: Bayesian networks
CPT: Conditional probability table
EM: Expectation maximisation
VBSA: Variance-based sensitivity analysis
AUROC: Area under the receiver operating characteristic curve
IQR: Interquartile Range
CRP: C-reactive protein
*E.coli*: *Escherichia coli*

## Footnotes

1 At a high level, the EM algorithm works in an iterative manner by initially choosing random (but valid) values for missing data, hence completing the dataset. This complete dataset is then used to perform a first parameterisation of the model (using the model’s existing CPTs as priors – in the present case, the priors were the expert elicited CPTs), which is then used to produce improved predictions of the missing data, which is in turn used to improve the model parameterisation again – with the process repeating until the model’s performance in predicting the data can be improved no further, converging to a final, locally optimal set of model parameters. (Convergence is guaranteed, as shown in (25).).

2 The specified antibiotic was classified as narrow (< = 3) or broader (> 3) according to published Antibiotic Spectrum Index (29). Narrow: Amoxicilin, Trimethoprim, Benzylpenicillin, Cefalexin, Cefazolin, Erythromicin. Broad: Amoxicillin + Clavulanic acid, Trimethoprim + Sulfamethoxazole, Co-trimoxazole, Amikacin, Cefepime, Cefotaxime, Ceftazidime, Ceftriaxone, Ciprofloxacin, Colistin, Ertapenem, Gentamicin, Meropenem, Moxifloxacin, Nitrofurantoin, Norfloxacin, Piperacillin + Tazobactam, Tobramycin, Vancomycin.

3 For the k-fold cross-validation, we chose k = 10 for its stable performance and efficient computational requirements [36]. Multiple values of k were investigated (k = 2, 5 and 20) and we found no notable difference in their performance in terms of mean log loss and AUROC.
