# Supplementary figures and images for: Urinary tract infections in children: building a causal model-based decision support tool for diagnosis with domain knowledge and prospective data
Source: BMC Med Res Methodol. 2022 Aug 8;22:218. doi: 10.1186/s12874-022-01695-6 (PMC9358867; doi:10.1186/s12874-022-01695-6)

### Additional file 1: Schematic of participant enrolment and data collection

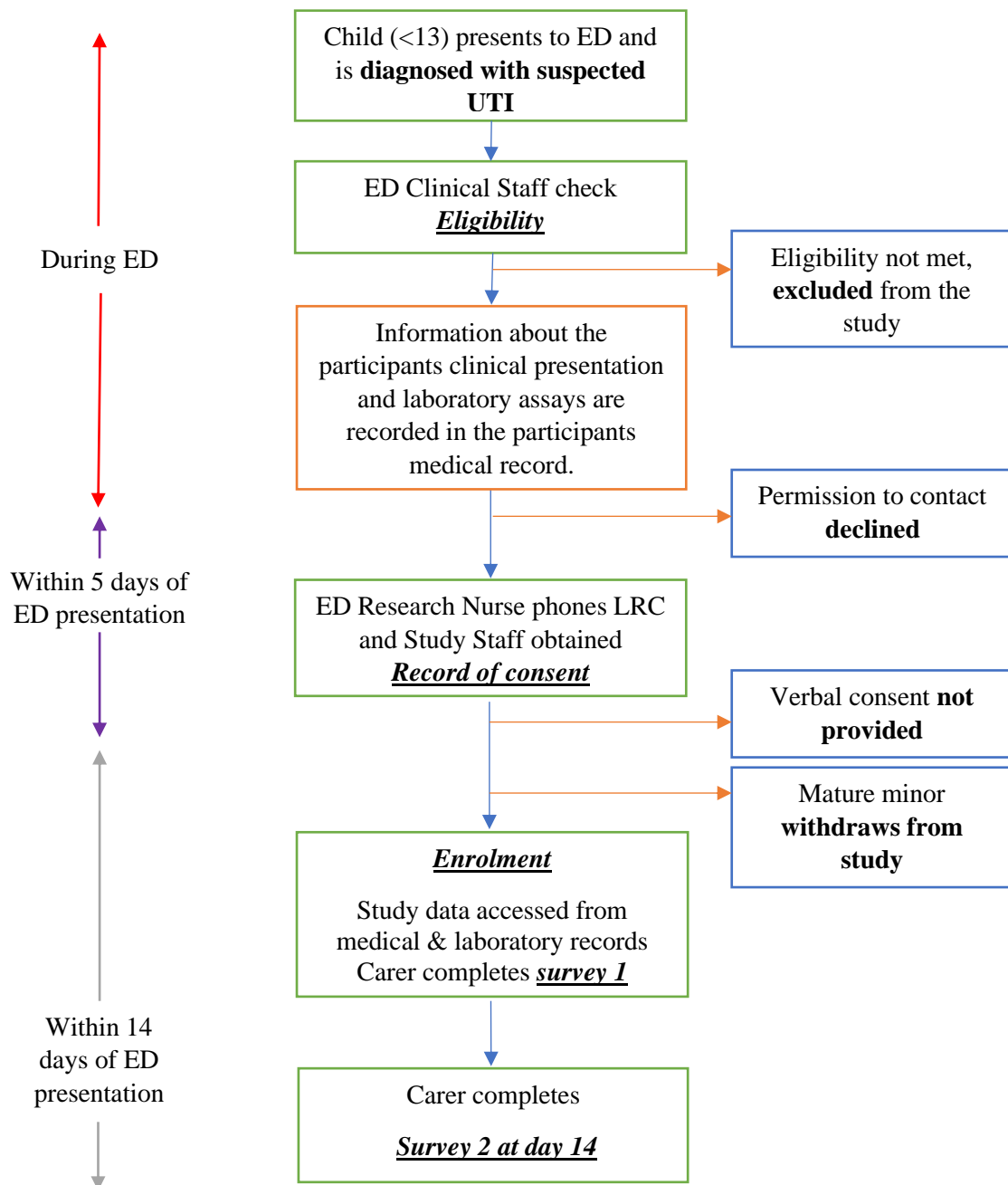

Supplement: Supplementary file 1 — Additional file 1. Schematic of participant enrolment and data collection. [file 12874_2022_1695_MOESM1_ESM.pdf]
