# Supplementary material for: Urinary tract infections in children: building a causal model-based decision support tool for diagnosis with domain knowledge and prospective data
Source: BMC Med Res Methodol. 2022 Aug 8;22:218. doi: 10.1186/s12874-022-01695-6 (PMC9358867; doi:10.1186/s12874-022-01695-6)
Supplement: Supplementary file 2 — Additional file 2. Parameterisation survey questions. [file 12874_2022_1695_MOESM2_ESM.pdf]

Additional file 2: The parameterisation survey questions

In this document we provided all survey questions used for the elicitation of parameters.

Q1. Risk of specimen contamination

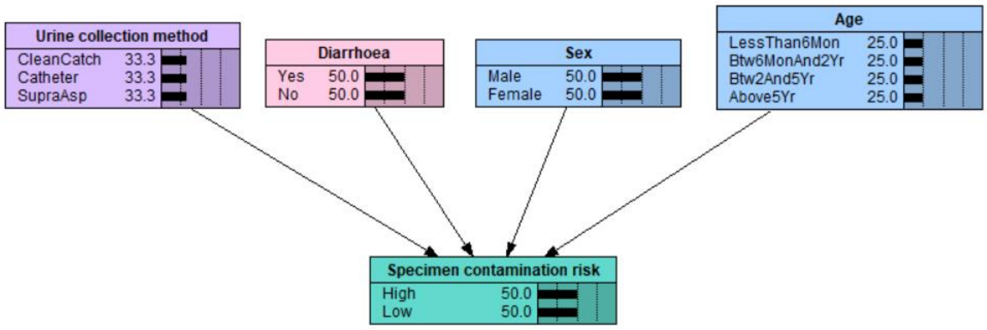

Consider the risk of a non-causative organism/s entering the urine specimen during the specimen collection process. In the model (as shown in the above figure), the **risk of specimen contamination** is influenced by **age**, **sex**, presence of **diarrhoea**, and **urine collection method**. Assuming *the same* colonisation status of each child's perineum/ external genitalia (i.e., type and density of organisms), how do the following factors increase or decrease the risk of specimen contamination from the baseline (as specified below)? E.g., x0.3, x2, x10, etc.

1a. **Age** and **sex**, assuming **clean catch** as the method of specimen collection.

**Age**	*Male*	*Female*
>=5yo	1 (baseline)	
2 to 5yo		
6mon to 2yo		
<6mon		

1b. Presence of **diarrhoea**, assuming **clean catch** as the method of specimen collection.

Absence	1 (baseline)
Presence	

1c. **Urine collection method**

Supra aspirate	1 (baseline)
Catheter	
Clean catch	

1d. Any further comments?

Q2. Propensity to UTI progression

Consider a child's risk of progressing to more severe disease manifestations given they have a UTI, e.g., developing kidney infection, or experiencing worsening severity of local or systemic inflammatory response, which can be further broken into two concepts: the **speed of progression**, and the **susceptibility to severity** – illustrated using the diagram below. Please note that these curves are illustrative, not exact.

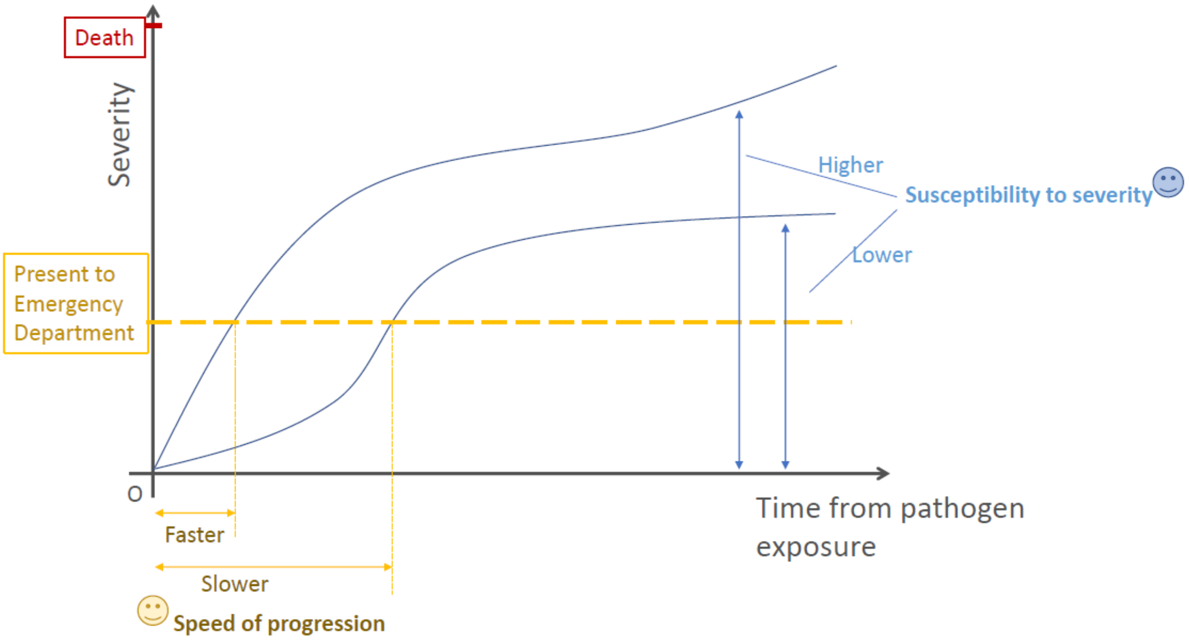

In the model, both the **speed of progression** and **susceptibility to severity** may be influenced by **age** and **UTI-relevant comorbidity** (such as VUR/anatomical abnormalities of the urinary tract). We now ask a series of questions on these two concept variables. Please provide your min, max, and best guess estimates for each question. Please note that the "min/max" should be plausible lower or upper values, e.g., 95th percentiles, not the extreme recordable value.

2a. **Speed of progression**

Assuming a baseline speed of progression (as specified below), how do the following factors increase or decrease the baseline? E.g., x0.3, x2, x10, etc.

Age

>=5yo	Speed of progression = 1 (baseline)		
**Estimate**	**Min**	**Max**	**Best**
2 to 5yo			
6mon to 2yo			
<6mon			

UTI-relevant comorbidity

No comorbidity	Speed of progression = 1 (baseline)		
**Estimate**	**Min**	**Max**	**Best**
With comorbidity			

Any further comments on the speed of progression?

E.g., will the impact be different for different comorbidities, pathogens?

2b. **Susceptibility to severity**

Assuming a baseline susceptibility to severity (as specified below), how do the following factors increase or decrease the baseline? E.g., x0.3, x2, x10, etc.

Age

>=5yo	Susceptibility to severity = 1 (baseline)		
**Estimate**	**Min**	**Max**	**Best**
2 to 5yo			
6mon to 2yo			
<6mon			

UTI-relevant comorbidity

No comorbidity	Susceptibility to severity = 1 (baseline)		
**Estimate**	**Min**	**Max**	**Best**
With comorbidity			

Any further comments on the susceptibility to severity?

E.g., will the impact be different for different comorbidities, pathogens?

Q3. Causative pathogen for UTI

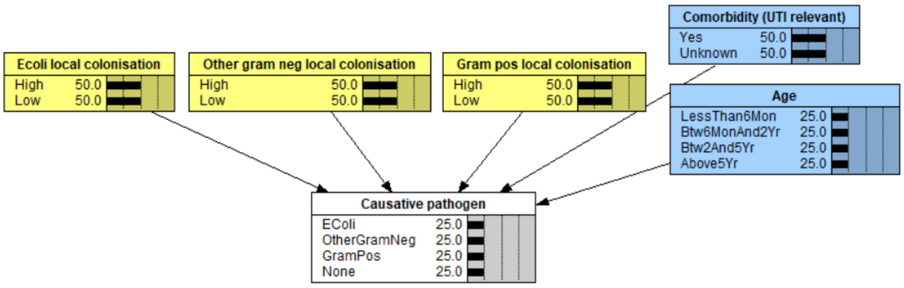

Colonisation of the perineum/ external genitalia by bacteria is assumed to predispose children to urinary tract infection (UTI). In the model, the probability of UTI with each causative pathogen (shown as **causative pathogen** in the above figure) is influenced by **age**, **local colonisation**, and **UTI-relevant comorbidity** (such as VUR/anatomical abnormalities of the urinary tract).

3a. Consider the PEA cohort, we enrolled children who presented to the Emergency Department (ED) at Perth Children's Hospital and were managed for presumed UTI (with an antibiotic prescription in the ED and a urine sample sent for laboratory investigation). These patients typically underwent urine dipstick in the ED. What do you estimate the probability (min, max, best guess) of true UTI in this cohort (prior to seeing the laboratory culture result)? Please note that the "min/max" should be plausible lower or upper values, not the extreme recordable value.

	True UTI, in %					
**Age**	*Boy*			*Girl*		
**Estimate**	**Min**	**Max**	**Best**	**Min**	**Max**	**Best**
>=5yo						
2 to 5yo						
6mon to 2yo						
<6mon						

3b. In the case of an otherwise healthy child with colonisation of the perineum/ external genitalia by all the following three groups of organisms: E.coli, other gram negatives, and gram positives. Note, we refer to gram positives that can potentially cause UTI, such as Enterococcus, rather than gram positives like Staph epidermidis which are unlikely cause UTI.

	Consider the E coli pathogenicity as baseline, could you please indicate the relative pathogenicity of others? E.g., x0.5, x3, etc.	Please comment if different pathogens affect the **speed of progression** and **susceptibility to severity** differently? If so, how?
E.coli	1 (baseline)	
Other gram negatives		
Gram positives (e.g., Enterococcus)		

3c. Any further comments?

Q4. Impact of exiting antibiotic use

4a. For modelling purpose, we have grouped antibiotics into two groups: narrow and broader, could you please review this grouping and suggest if any antibiotic should be grouped differently? Please feel free to add new group/s.

Narrow: Amoxicilin, Amoxicillin + clavulanic acid, Trimethoprim, Trimethoprim + Sulfamethoxazole, Benzylpenicillin, Cefalexin, Cefazolin, Co-trimoxazole, Erythromicin

Broader: Amikacin, Cefepime, Cefotaxime, Ceftazidime, Ceftriaxone, Ciprofloxacin, Colistin, Ertapenem, Gentamicin, Meropenem, Moxifloxacin, Nitrofurantoin, Norfloxacin, Piperacillin + Tazobactam, Tazocin, Tobramycin, Vancomycin

Any further comments?

4b. Given a UTI, the successful detection of the causative pathogen of the UTI in laboratory can be influenced if the patient has been on antibiotic when they came to the ED where the urine sample was taken. Presumably this is largely affected by the antimicrobial susceptibility pattern of the pathogen which can variable by different subgroups, so please consider an average community-acquired case in 2019-2020.

Under the following scenarios, please provide your min, max, and best guess estimates for each question. Please note that the "min/max" should be plausible lower or upper values, not the extreme recordable value. (Please feel free to refer to your experience of treating UTI in adults.)

Consider a UTI caused by E.coli

	Probability of positive culture of E.coli		
**Estimate**	**Min**	**Max**	**Best guess**
Not on abx			
On narrow abx			
On broader abx			
Pls feel free to add more groups			

Consider a UTI caused by other gram negative bacteria

	Probability of positive culture of other gram neg		
**Estimate**	**Min**	**Max**	**Best guess**
Not on abx			
On narrow abx			
On broader abx			

Consider a UTI caused by gram positive bacteria (e.g., Enterococcus)

	Probability of positive culture of gram pos		
**Estimate**	**Min**	**Max**	**Best guess**
Not on abx			
On narrow abx			
On broader abx			

Any further comments?
