# Supplementary material for: Urinary tract infections in children: building a causal model-based decision support tool for diagnosis with domain knowledge and prospective data
Source: BMC Med Res Methodol. 2022 Aug 8;22:218. doi: 10.1186/s12874-022-01695-6 (PMC9358867; doi:10.1186/s12874-022-01695-6)
Supplement: Supplementary file 3 — Additional file 3. The Expert DAG and variable dictionary. [file 12874_2022_1695_MOESM3_ESM.pdf]

Additional file 3: The Expert DAG and variable dictionary

In this document we provided the structure of the Expert DAG v11.1 (Figures C1) and its dictionary (Table C1).

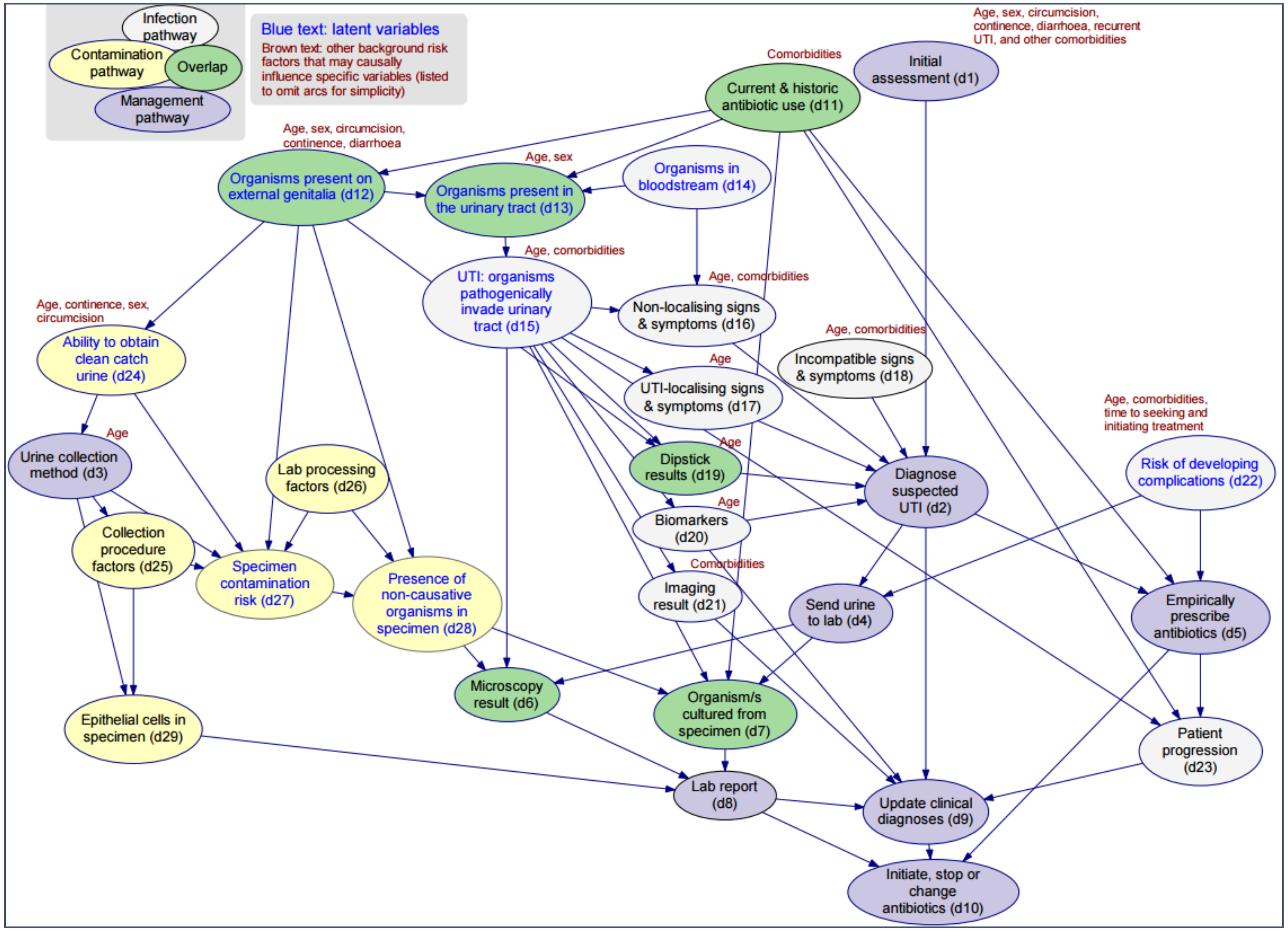

Figure C1. The Expert DAG v11.1, as provided in the main manuscript (Figure 1).

Table C1. Variable dictionary of the Expert DAG v11.1.

Variable name	Description	Parent nodes in the Expert DAG	How affected by parent nodes
**Infection pathway**			
**UTI: organisms pathogenically invade urinary tract (d15)**	Pathogenic organism infecting the urinary tract and causing disease. This is a latent concept describing a UTI in terms of its true pathophysiological state.	Organisms present in the urinary tract (d13), age, comorbidities	As organisms must first enter the urinary system before causing an infection. As age and comorbidities (such as structural urinary system or immune system abnormalities) impact susceptibility to disease and therefore may directly drive the development of a true UTI.
**UTI-localising signs & symptoms (d17)**	Patient features that are localised to indicate the urinary tract.	UTI: organisms pathogenically invade urinary tract (d15), age	UTI-specific signs and symptoms such as dysuria can be driven by the presence of a true UTI and age. The influence of age is primarily due to differences in verbal communication of pain and discomfort.
**Non-localising signs & symptoms (d16)**	Patient features that can indicate UTI but are not specific to a UTI.	UTI: organisms pathogenically invade urinary tract (d15), organisms in bloodstream (d14), age, comorbidities	The observation of non-specific symptoms such as fever can be influenced by age and comorbidities. These signs and symptoms can indicate an increased probability of a true UTI, but to a lesser extent when compared with UTI-specific signs and symptoms. If organisms are present in bloodstream, this can also drive the presentation of non-specific signs & symptoms but may not be discernible from the symptoms of a UTI.
**Incompatible signs & symptoms (d18)**	Patient features that are unlikely caused by UTI.	Age, comorbidities	Age and comorbidities may influence the probability of incompatible signs & symptoms due to differing susceptibility to other diseases.

Variable name	Description	Parent nodes in the Expert DAG	How affected by parent nodes
**Organisms in bloodstream (d14)**	Pathogenic bacteria in blood stream. This is a latent concept distinct from a diagnosis of bacteraemia.<br>Blood infections seeding the kidneys are rare, yet possible, for example fungal infections in immunosuppressed population. Most positive blood cultures in patients with UTI represent invasion of the blood stream from an infection starting in the urinary tract (urosepsis).	None	NA
**Biomarkers (d20)**	Laboratory test result providing insight on the clinical picture of the patient. This may include C-reactive protein or a full blood count.	UTI: organisms pathogenically invade urinary tract (d15), age	Biomarkers tested may produce abnormal results driven by the presence or absence of a true UTI. These include inflammatory markers such as CRP and white blood cell count. The influence of age is primarily due to differences in the maturity of the child’s immune system thus it’s response in the form of elevated biomarkers.
**Imaging result (d21)**	Radiology imaging results, such as ultrasound, which provide information on potential pathologies of the urinary system.	UTI: organisms pathogenically invade urinary tract (d15), comorbidities	An imaging result, describing abnormal urinary system features suggestive of a UTI for example, is directly influenced by the presence of a UTI. Chronic urinary tract or kidney problems can also be seen via imaging.
**Risk of developing complication (d22)**	This is a latent concept that collectively describes the risk of progressing to a severe or complicated state of disease.	Age, comorbidities, time to seeking and initiating treatment	Susceptibility to complications of disease is driven by a patient’s age and the presence of comorbidities such as urinary system and immune system abnormalities which can cause a more severe UTI. The risk of developing complications is also driven by the time to seeking medical care. For example, a patient may not be assessed by a clinician within an appropriate time and therefore the patient will present to the hospital with a complicated and severe UTI.

Variable name	Description	Parent nodes in the Expert DAG	How affected by parent nodes
**Patient progression (d23)**	The patient's state after time and clinical management.	UTI: organisms pathogenically invade urinary tract (d15), current & historic antibiotic use (d11), empirically prescribe antibiotics (d5)	A patient's progress is directly influenced by the presence of a true UTI and their response to clinical management such as the prescription of antibiotics.
**Contamination pathway**			
**Ability to obtain clean catch urine (d24)**	Access to a sterile urine specimen (without use of invasive methods). This is a latent term used to describe a patient's seamless or difficult ability to provide a sterile urine specimen free from potential contaminants.	Organisms present on external genitalia (d12), age, continence, sex, circumcision	Age and continence can influence this due to an individual's ability to follow instructions for providing access to clean urine. The presence of bacteria on the external genitalia can influence access to sterile urine as well as sex and circumcision due to the differences in the proximity of bacterial flora to the external opening of the urethral.
**Urine collection method (d3)**	Method of urine sample collection chosen by the clinician.	Age	The choice of collection method is driven by an ability to produce a clean urine sample and age due to the patient ability to follow instructions required for particular urine specimens.
**Collection procedure factors (d25)**	Factors relating to collection of the specimen such as number of attempts to collect the specimen, time from cleaning the perineal area to collection of the urine specimen and collection of the specimen in accordance with the specified procedure.	Urine collection method (d3)	Specimen collection factors are driven by the method of urine collection.

Variable name	Description	Parent nodes in the Expert DAG	How affected by parent nodes
**Lab procedural factors (d26)**	Factors relating to laboratory processing of the urine sample that increase the risk of contamination from the time the urine arrive in the lab to the final reporting. This can include delays in sample processing and refrigeration.	None	NA
**Specimen contamination risk (d27)**	This is a latent term expressing all factors contributing to contamination.	Organisms present on external genitalia (d12), ability to obtain clean catch urine (d24), urine collection method (d3), collection procedure factors (d25), lab processing factors (d26)	This is driven by all factors that introduce or concentrate non-causative organism from collection to lab processing.
**Presence of non-causative organisms in specimen (d28)**	Presence and concentration of microorganisms in the urine specimens that are not causing an urinary tract infection.	Organisms present on external genitalia (d12), specimen contamination risk (d27), lab processing factors (d26)	The presence of non-causative organisms is driven by the presence of organisms the external genitalia and the specimen contamination risk. The lab procedural factors (e.g., longer delays) can allow these organisms to proliferate to a higher concentration.
**Epithelial cells in specimen (d29)**	Epithelial cells entering the urine specimen during collection and reported by the laboratory.	Urine collection method (d3), collection procedure factors (d25)	The presence of epithelial cells in the specimen is driven by urine collection methods and collection procedure factors that cause epithelial cells to enter the urine specimen.
**Overlap**			
**Background risk factors**	Many known risk factors that increase the risk of UTI, complication and contamination, such as age, sex at birth, circumcision, recurrent UTI, immobility, continence, diarrhoea and other comorbidities (a complex story which is not the focus of this paper).	None	NA

Variable name	Description	Parent nodes in the Expert DAG	How affected by parent nodes
**Current and historic antibiotic use (d11)**	A patient's receipt of antibiotics at the time of presentation or recently prior to assessment. Frequency of historic use is also captured within this node.	Comorbidities	Antibiotic use may be driven by some comorbidities such as congenital urological abnormalities.
**Organisms present on external genitalia (d12)**	Organism present on external genitalia.	Current & historic antibiotic use (d11), age, sex, circumcision, continence, diarrhoea	Organism presence, type and amount are influenced by a patient's age, sex, whether they are circumcised, their level of continence and current or recent diarrhoea. Current or historic antibiotic use can influence the type and amount of bacteria present.
**Organisms present in urinary tract (d13)**	An organism entering the urinary tract. This is the precursor to a UTI.	Organisms present on external genitalia (d12), current & historic antibiotic use (d11), age, sex	This is driven by organisms entering the urinary tract via the blood stream during bacteraemia (although rare) or ascending from the external genitalia. A patient's sex and gender can influence this process due to anatomical differences and susceptibility to disease. Historic or current antibiotic treatment may also drive the presence of organisms.
**Dipstick results (d19)**	Urine analysis/testing available at the point of care.	UTI: organisms pathogenically invade urinary tract (d15), organisms present on external genitalia (d12), age	Dipstick results are directly influenced by the presence or absence of a true UTI, particularly leukocyte esterase and nitrites. Nitrites produced by the colonising gram negative bacteria can be detected from the urine sample even the bacteria is not causing the UTI. The amount of leukocyte esterase can be influenced by age-specific immune response.
**Microscopy result (d6)**	The presence of white blood cells, red blood cells and bacteria detected by microscopic investigation of the urine sample.	UTI: organisms pathogenically invade urinary tract (d15), presence of non-causative organisms in specimen (d28), send urine to lab (d4)	The presence of bacteria can be due to either the proliferating bacteria that causes a true UTI, or the presence of non-causative organisms in the specimens, or both. The presence of white and red blood cells is typically driven by inflammation or damage caused by a true UTI. Urine microscopy can only be reported if the urine is sent to the lab.

Variable name	Description	Parent nodes in the Expert DAG	How affected by parent nodes
**Organism/s cultured from specimen (d7)**	Growth of microorganisms from a urine specimen.	UTI: organisms pathogenically invade urinary tract (d15), presence of non-causative organisms in specimen (d28), current & historic antibiotic use (d11), send urine to lab (d4)	The presence, type and amount of organism is driven by the presence of causative or non-causative organisms in the urine specimen, recent antibiotic use, which may change the bacteria grown and their antibiotic susceptibility profiles. Culture can only occur if the decision is made to send the urine to the lab.
**Management pathway**			
**Initial assessment (d1)**	Prior belief of a UTI based on the assessment of the patient's history and background risk factors.	Age, sex, circumcision, continence, diarrhoea, recurrent UTI, and other comorbidities	This belief is driven to a range of factors including a patient's age, sex, circumcision, diarrhoea and other comorbidities (including those that pre-dispose children to recurrent urinary tract infections).
**Diagnose suspected UTI (d2)**	Diagnosis of a suspected urinary tract infection by the treating clinician.	Initial assessment (d1), non-localising signs & symptoms (d16), UTI-localising signs & symptoms (d17), incompatible signs & symptoms (d18), dipstick results (d19), biomarkers (d20)	Following the initial assessment, the diagnosis made by the treating clinician is formed by their further consultation and investigation including those signs and symptoms that match the picture of a UTI (e.g. dysuria) and those less specific to a UTI (e.g. fever). Symptoms such as rhinorrhoea that are not driven by a true UTI but may be present at the time of clinical assessment can reduce the probability a clinician's suspect the presence of a UTI during the initial assessment. If a dipstick is performed and biomarker results (e.g. CRP) are returned abnormal this may also drive the suspicion of a UTI.
**Send urine to lab (d4)**	Clinical decision to send the urine specimen to the laboratory for culture.	Diagnose suspected UTI (d2), risk of developing complications (d22)	This is driven by the clinician's suspicion of a UTI and the patient's susceptibility to complications as it is more important to ensure correct management when the risk is higher.
**Empirically prescribe antibiotic (d5)**	Clinical decision to prescribe (empiric) antibiotics in the ED.	Diagnose suspected UTI (d2), risk of developing complications (d22),	This decision is driven by the diagnosis of a UTI, previous or current antibiotic use as a clinician may choose to change or keep the antibiotic type, and susceptibility to

Variable name	Description	Parent nodes in the Expert DAG	How affected by parent nodes
		current & historic antibiotic use (d11)	complications influences both the decision to prescribe and the type of antibiotic.
**Lab report (d8)**	Interpretation and reporting of the urine specimen by the laboratory.	Organism/s cultured from specimen (d7), microscopy result (d6), epithelial cells in specimen (d29)	A urine culture report is driven thresholds and reporting rules according to the organism(s) cultured from the specimen, the microscopy results and the presence and quality of epithelial cells in the specimen.
**Update clinical diagnoses (d9)**	Clinical diagnosis based on updated information.	Diagnose suspected UTI (d2), lab report (d8), biomarkers (d20), imaging result (d21), patient progression (d23)	The diagnosis is influenced by the initial suspicion of a UTI and additional information gained from the laboratory report, biomarker and imaging results and the progress the patient has made with clinical management that may align with a UTI or indicate an alternative illness.
**Change, initiate or stop antibiotics (d10)**	A clinician’s decision to change (including stop) or initiate antibiotics to treat a urinary tract infection based on the receipt of further information	Lab report (d8), update clinical diagnoses (d9), empirically prescribe antibiotics (d5)	The information (if available) driving this decision can include the laboratory report with antibiotic susceptibility results, the clinician’s updates clinical diagnoses and whether the patient was empirically prescribes antibiotics.
