# Supplementary material for: Urinary tract infections in children: building a causal model-based decision support tool for diagnosis with domain knowledge and prospective data
Source: BMC Med Res Methodol. 2022 Aug 8;22:218. doi: 10.1186/s12874-022-01695-6 (PMC9358867; doi:10.1186/s12874-022-01695-6)
Supplement: Supplementary file 4 — Additional file 4. List of changes when converting the Expert DAG to the Applied BN. [file 12874_2022_1695_MOESM4_ESM.pdf]

Additional file 4: List of changes when converting the Expert DAG to the Applied BN

In this document we summarised the major considerations when converting the Expert DAG to the Applied BN. Conversion of the Expert DAG took into consideration: how a particular variable is relevant to the applied BN's purpose; how it could be matched to available data; and how it could help simplify parameterisation or computational workload. This frequently involved simplifications by removing and merging variables, as well expansions by splitting and adding variables. Figure D1 illustrates an example list of decisions made on whether to keep a DAG variable in the BN. We also provided a full list of changes occurred during the conversion from the Expert DAG v11.1 to the Applied BN v2.2 (Table D1).

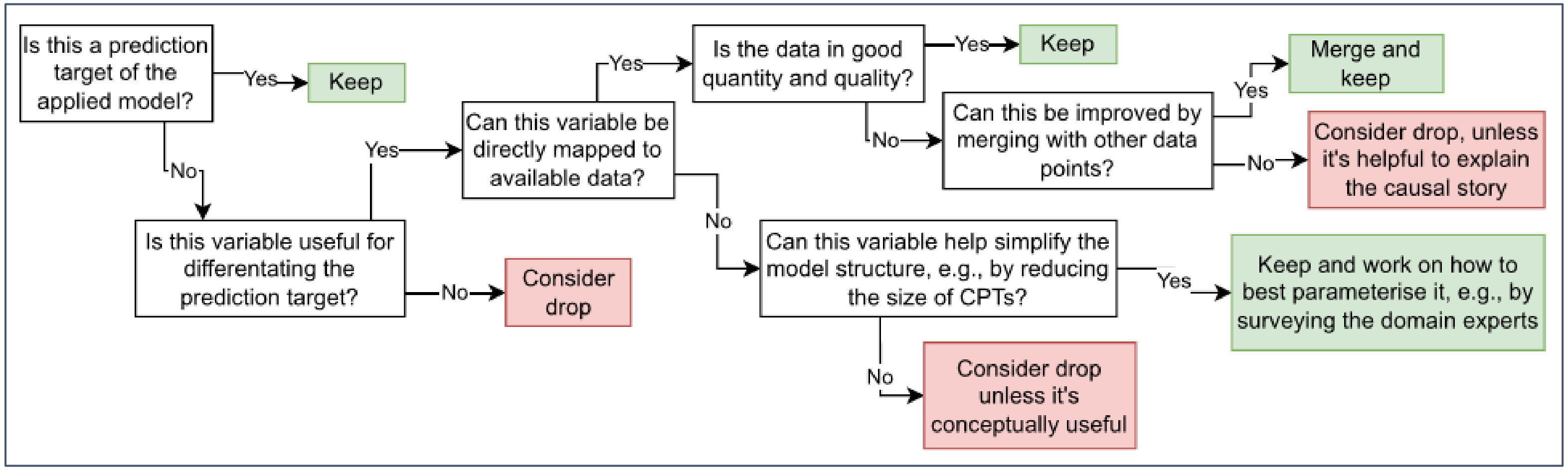

Figure D1. An example procedure of deciding on whether to keep a DAG variable in the Applied BN.

Table D1. Structural changes occurred during our conversion from the Expert DAG v11.1 to the Applied BN v2.2 and their underlying rationale.

**Changes**	**Expert DAG**	**Applied BN**
Route of UTI and causative pathogens	Organisms present on external genitalia (d12) and organisms in bloodstream (d14) are two possible routes that can lead to organisms present in the urinary tract (d13) and subsequently UTI (d15).	Acquiring UTI from bloodstream (d14) is rare, thus was dropped for simplification. Grouping d12 and d13 together as local colonisation, which approximates a broader area where presence of organism is relevant (possible to cause UTI). Splitting a general organism concept into three specific organism groups (b7-9), which are the primary predication targets of the model.
Ability to obtain clean catch urine	Explicitly described as a concept that can be influenced by age and sex etc. (d24), and subsequently drives urine collection methods (d3), contamination risk (d27).	This concept node is removed to simplify the parameterisation need, instead, relevant background factors directly go into urine collection methods (b13) and contamination risk (b15) to maintain the associations.
Presence of non-causative organism in specimen	An intermediate step that explicitly describes how (non-causative) colonising organisms may be present in specimen thus isolated (d28).	This intermediate node was removed for simplicity, as a result, the local colonisations (b7-b9) directly influence the culture results (b17-b19).
Collection, lab procedure factors	Described using d25-26 for completeness of the causal story.	Dropped as considered not highly influential by the domain experts, as well as with insufficient data for investigation.
Background risk factors	Simplified as brown text.	Key ones explicitly included as variables/nodes: age group (b1), UTI-relevant comorbidity (b3), sex (b2) and diarrhoea (b4).
Culture results	Summarised as a single node (d7)	Organism group specific nodes b17-19
Symptoms and signs	Single node was used to summarise a group of signs and symptoms, including those are UTI localising (d17), non-localising (d16), and incompatible with UTI (d18).	Specific signs and symptoms are described using separate nodes/variables with certain interactions among the signs and symptoms (b26-37). Looking for differential effects of different signs and symptoms. Observations can be made from the presenting patients, entered as input variables when applying the model.
Dipstick	Included as a summary node (d19)	Expanded for differential effects based on data availability (b20-21).
Biomarkers	Included as a summary node (d20)	Expanded for differential effects based on data availability (b23-25).
Microscopy result	Included as a summary node (d6)	Expanded for differential effects based on data availability (b16, b22).
Initial assessment, diagnosis of suspected UTI	d1-2, clinical diagnosis based on evidence available to treating doctor at the point of care.	Suspected UTI was an inclusion criteria for the PEA study cohort, thus these variables were removed. As a result, we are not able to explore how such clinical diagnosis was made.

**Changes**	**Expert DAG**	**Applied BN**
Send urine to lab	d4	This was an inclusion criteria for the PEA study cohort of suspected UTI, thus this variable is removed. As a result, we are not able to explore how the urine test decision was made.
Lab report	d8, included to summarise results on microscopy results (d6), epithelial cells (d29) and culture results (d7).	Dropped as the microscopic analysis (b16, b22), epithelial cells (b14), and culture results (b17-19) were explicitly included.
Propensity of UTI Progression	Risk of developing complications (d22)	This variable was first divided into two concepts: susceptibility to UTI progression (b5) and current UTI severity (b11). After further workshop discussion, b5 and b11 were merged as one variable (for structure simplicity) and renamed as current clinical phenotype (b11).
Patient outcome of the current epsiode	Described using patient progression (d23), update clinical diagnoses (d9), and initiate, stop or change antibiotics (d10).	All dropped as they are out of the scope of the current application.
