# Supplementary material for: Urinary tract infections in children: building a causal model-based decision support tool for diagnosis with domain knowledge and prospective data
Source: BMC Med Res Methodol. 2022 Aug 8;22:218. doi: 10.1186/s12874-022-01695-6 (PMC9358867; doi:10.1186/s12874-022-01695-6)
Supplement: Supplementary file 5 — Additional file 5. Full structure of the Applied BN and the BN dictionary. [file 12874_2022_1695_MOESM5_ESM.pdf]

Additional file 5: Full structure of the Applied BN and the BN dictionary

In this document we provided the detailed structure of the Applied BN v2.2 include a high-level structure (Figure E1) and 4 submodels (Figures E2-E5) and the variable dictionary of this model (Table E1).

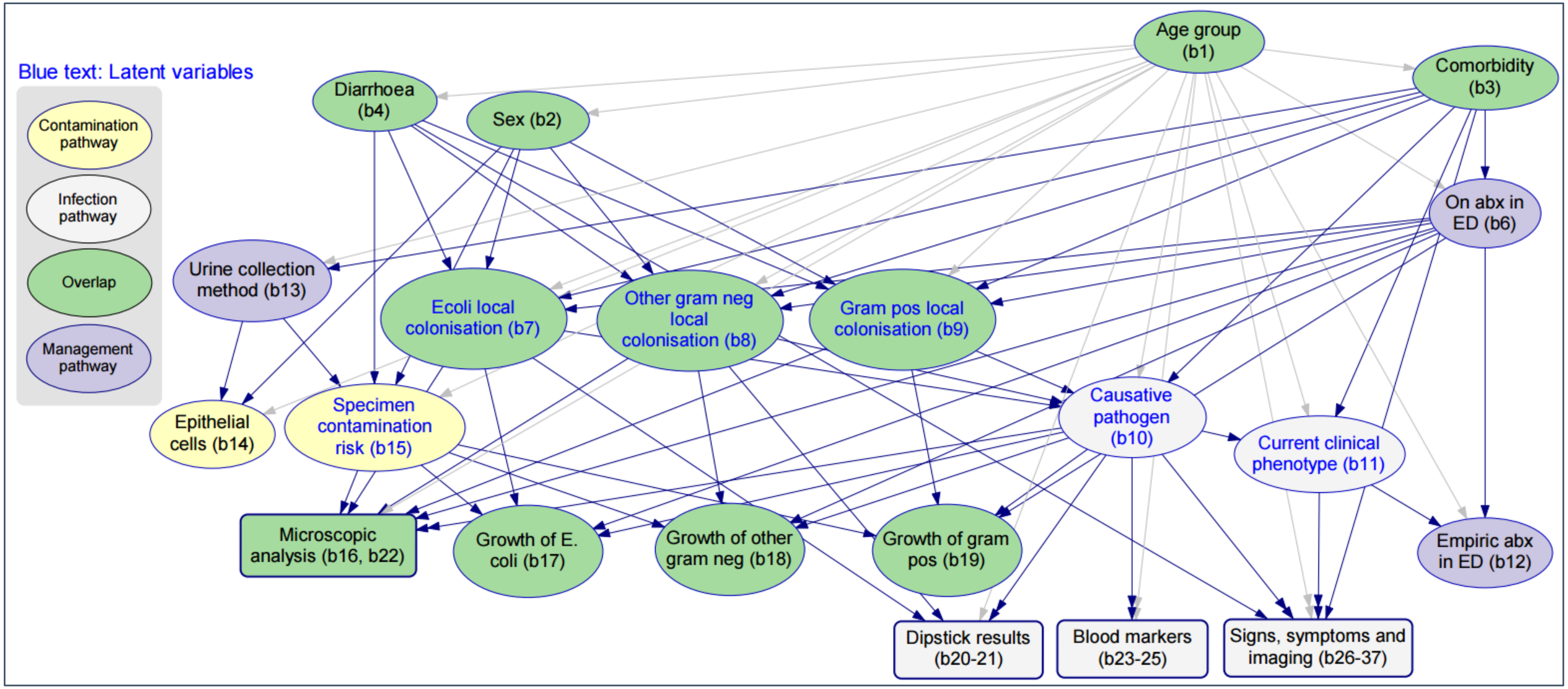

Figure E1**. **The high-level structure of the Applied BN v2.2, as provided in the main manuscript (Figure 2, bottom panel).

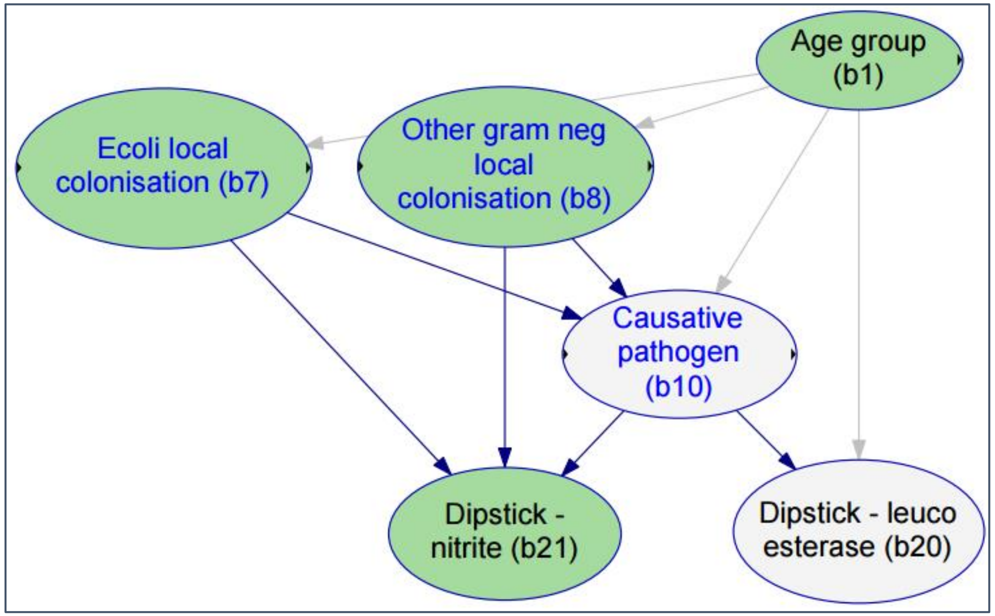

Figure E2. The local structure of dipstick results submodel (b20-21) with external connections (b1, b7-8, b10).

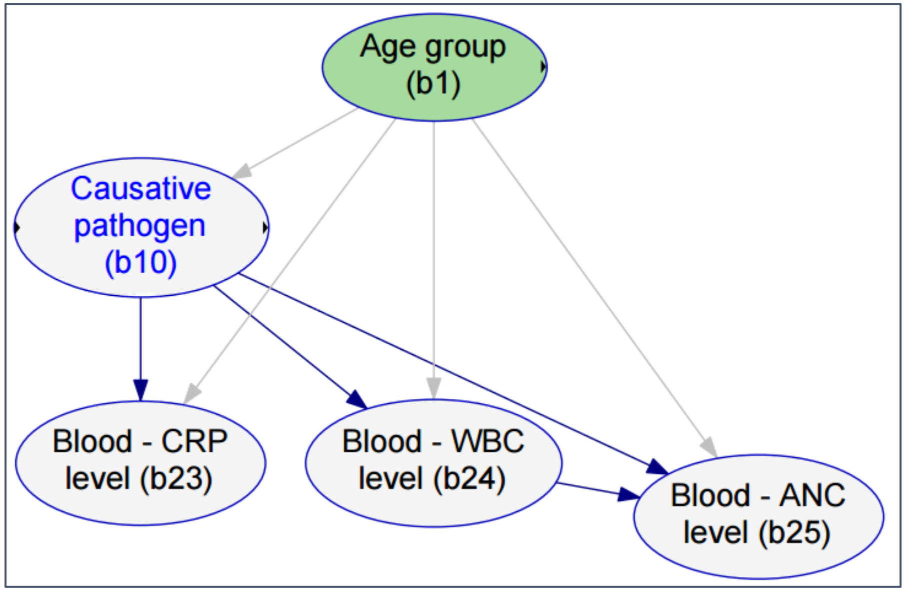

Figure E3 The local structure of microscopic analy (b23-25) with external connections (b1, b10).

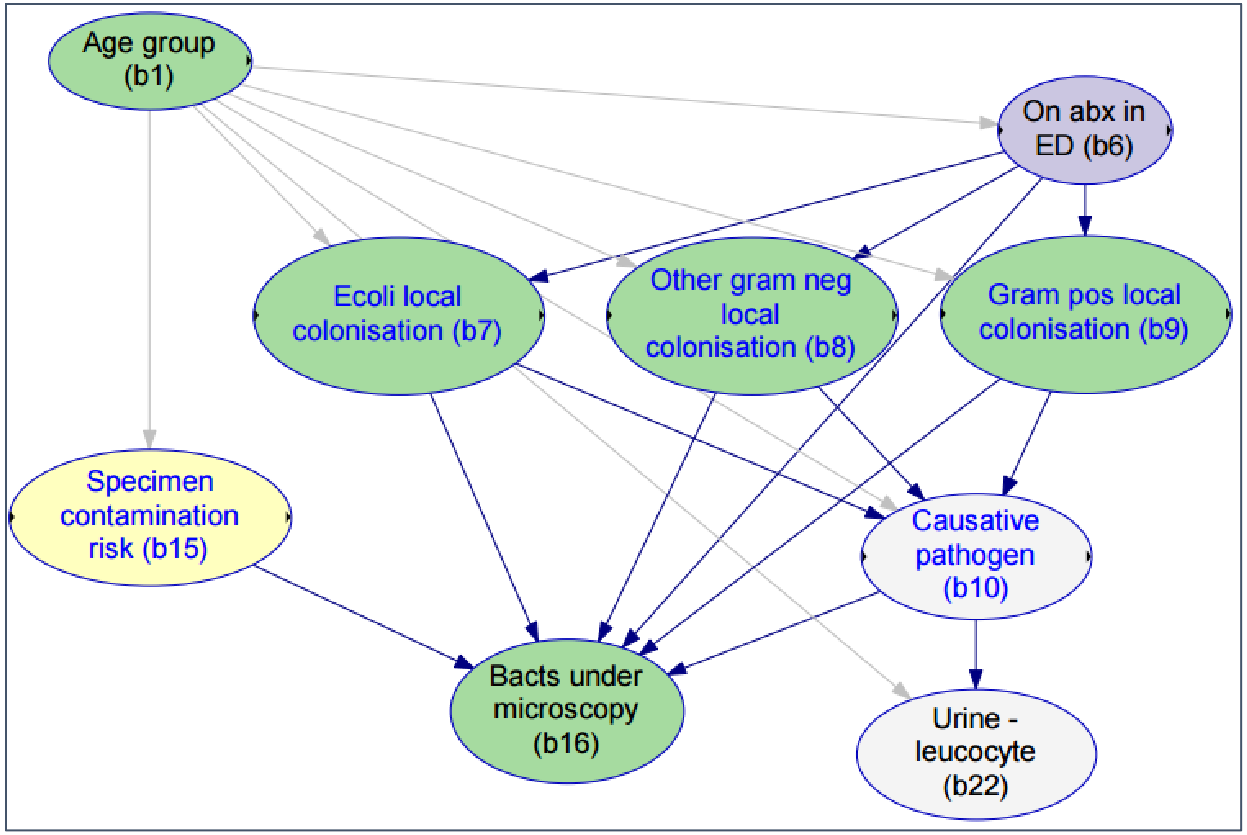

Figure E4 The local structure of microscopic analysis submodel (b16, b22) with external connections (b1, b6, b7-10, b15).

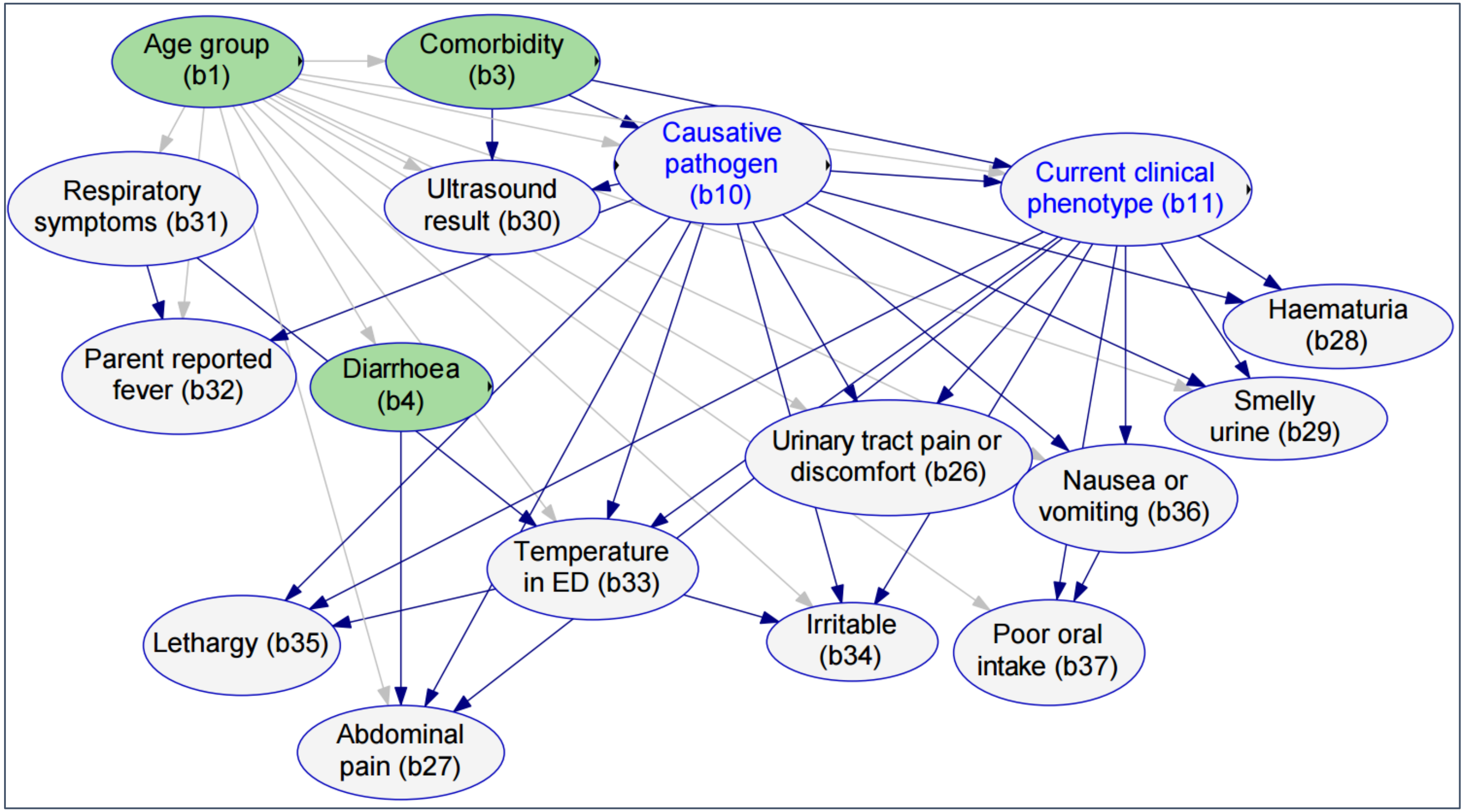

Figure E5 The local structure of signs, symptoms and imaging (b26-b37) with external connections (b1, b3-4, b10, b11). Of note, although the history of diarrhoea (b4) can be a symptom caused by UTI, we modelled it as an important possible cause of UTI therefore excluded from the signs, symptoms and imaging submodel. However, the presence of diarrhoea may lead to reported abdominal pain, thus shown here as an external connection.

Table E1 The variable dictionary of the Applied BN v2.1, and how the variables correspond to the Expert DAG v11.1.

vid	Variable label	Definition	States	Parents	Corresponding node/s in the Expert DAG v11	Status
b1	**Age group**	Age group. All children under 13yo as an inclusion criteria for the PEA Study.	*LessThan6Mon, Btw6MonAnd2Yr, Btw2And5Yr, Above5Yr*	None	Simplified as brown text	Observable
b2	**Sex**	Sex at birth.	*Female, Male*	Age group	Simplified as brown text	Observable
b3	**Comorbidity**	History of UTI or a history of urinary tract problems, either recorded within their medical notes or reported by their carers in the PEA study survey.	*Reported, Unknown*	Age group	Simplified as brown text	Observable
b4	**Diarrhoea**	History of diarrhoea. Although this can be a symptom caused by UTI, we modelled it as an important possible cause of UTI rather than caused by.	*Yes, No*	Age group	Simplified as brown text	Observable
b6	**On abx in ED**	On antibiotics upon presentation to ED, if yes, the specified antibiotic was classified as narrow (<=3) or broader (>3) according to published Antibiotic Spectrum Index (Gerber et al., 2017)[1].	*Narrow, Broader, No*	Age group, comorbidity	d11	Observable
b7	**E.coli local colonisation**	The level of colonisation of the perineum/ external genitalia by E. coli., this is assumed to predispose children to E. coli UTI.	*High, Low*	Age group, sex, comorbidity, diarrhoea, on abx in ED	d12, d13	Latent
b8	**Other gram neg local colonisation**	The level of colonisation of the perineum/ external genitalia by non-E. coli gram negative bacteria, this is assumed to predispose children to other gram negative UTI.	*High, Low*	Age group, sex, comorbidity, diarrhoea, on abx in ED	d12, d13	Latent

vid	Variable label	Definition	States	Parents	Corresponding node/s in the Expert DAG v11	Status
b9	**Gram pos local colonisation**	The level of colonisation of the perineum/ external genitalia by gram positive bacteria, this is assumed to predispose children to gram positive UTI.	*High, Low*	Age group, sex, comorbidity, diarrhoea, on abx in ED	d12, d13	Latent
b10	**Causative pathogen**	The pathogenic organism infecting the urinary tract and causing disease.	*EColi, OtherGramNeg, GramPos, None*	Age group, comorbidity, E.coli local colonisation, other gram neg local colonisation, gram pos local colonisation	d15	Latent
b11	**Current clinical phenotype**	This was introduced as a summary node of patient presentation phenotypes based on signs and symptoms relevant to UTI.	*Type 1, Type 2, Type 3*	Age group, comorbidity, causative pathogen	d22	Latent[2]
b12	**Empiric abx in ED**	Empiric antibiotic prescription received during the ED presentation. This is an inclusion criteria for the PEA Study.	*Narrow, Broader*	On abx in ED, current clinical phenotype, age group	d5	Observable
b13	**Urine collection method**	Method of urine sample collection chosen by the clinician.	*CleanCatch, SupraAsp, Catheter*	Age group, comorbidity	d3	Observable
b14	**Epithelial cells**	Assessment of epithelial cells via microscopic or automated analysis.	*Moderate, Low*	Age group, sex, urine collection method	d29	Observable
b15	**Specimen contamination risk**	This latent concept refers to the risk of a non-causative organism/s entering the urine specimen during the specimen collection process.	*High, Low*	Age group, sex, diarrhea, urine collection method	d27	Latent

vid	Variable label	Definition	States	Parents	Corresponding node/s in the Expert DAG v11	Status
b16	**Bacts under microscopy**	Assessment of bacteria via microscopic analysis.	*Many, Moderate, Few, NotSeen*	Causative pathogen, on abx in ED, specimen contamination risk, E.coli local colonisation, other gram neg local colonisation, gram pos local colonisation	d6	Observable
b17	**Growth of E.coli**	The isolation of E.coli from the urine specimen.	*Positive, Negative*	E.coli local colonisation, specimen contamination risk, on abx in ED, causative pathogen	d7	Observable
b18	**Growth of other gram neg**	The isolation of non-E.coli gram negative bacteria from the urine specimen.	*Positive, Negative*	Other gram neg local colonisation, specimen contamination risk, on abx in ED, causative pathogen	d7	Observable
b18	**Growth of gram pos**	The isolation of gram positive bacteria from the urine specimen.	*Positive, Negative*	Gram pos local colonisation, specimen contamination risk, on abx in ED, causative pathogen	d7	Observable
b20	**Dipstick - leuco esterase**	Leukocyte esterase detected on urine dipstick.	*High, Moderate, Low, NotDetected*	Causative pathogen, age group	d19	Observable
b21	**Dipstick - nitrite**	Nitrite detected on the urine dipstick.	*Detected, NotDetected*	Causative pathogen, E.coli local colonisation, other gram neg local colonisation	d19	Observable

vid	Variable label	Definition	States	Parents	Corresponding node/s in the Expert DAG v11	Status
b22	**Urine - leucocyte**	The assessment of leucocytes level in the urine specimen via microscopic analysis.	*High*, *Moderate*, *Low*	Causative pathogen, age group	d6	Observable
b23	**Blood - WBC level**	Leukocyte counts in blood.	*Above18*, *Btw10And18*, *Below10*, *NotDone*	Causative pathogen, age group	d20	Observable
b24	**Blood - CRP level**	C-Reactive protein in blood.	*Above70*, *Btw15And70*, *Below15*, *NotDone*	Causative pathogen, age group	d20	Observable
b25	**Blood - ANC level**	Absolute neutrophil counts in blood.	*Above15*, *Btw8And15*, *Below8*, *NotDone*	Causative pathogen, age group, blood - WBC level	d20	Observable
b26	**Urinary tract pain or discomfort**	Genital pain (or discomfort), dysuria, and urinary tract relevant discomfort based on free text in medical notes recorded during ED presentation.	*Yes*, *Unknown*	Causative pathogen, current clinical phenotype, age group	d17	Observable
b27	**Abdominal pain**	Abdominal pain in medical notes recorded during ED presentation.	*Yes*, *Unknown*	Causative pathogen, current clinical phenotype, age group, diarrhea	d16, d17	Observable
b28	**Haematuria**	Haematuria in medical notes recorded during ED presentation.	*Yes*, *Unknown*	Causative pathogen, current clinical phenotype	d17	Observable
b29	**Smelly urine**	Foul smelling urine in medical notes recorded during ED presentation.	*Yes*, *Unknown*	Causative pathogen, current clinical phenotype, age group	d17	Observable

vid	Variable label	Definition	States	Parents	Corresponding node/s in the Expert DAG v11	Status
b30	**Ultrasound result**	Result of ultrasound investigation. Abnormal ultrasound result was defined if there were features suggestive of pyelonephritis, renal abscess, cystitis or other UTI evidence.	*Abnormal, unknown, NotDone*	Age group, causative pathogen, comorbidity	d21	Observable
b31	**Respiratory symptoms**	Respiratory symptoms in medical notes recorded during ED presentation.	*Yes, No*	Age group	d18	Observable
b32	**Parent reported fever**	Parent reported fever in medical notes recorded during ED presentation.	*Yes, No*	Causative pathogen, age group, respiratory symptoms	d16	Observable
b33	**Temperature in ED**	Temperature (degrees celsius) recorded in ED.	*Abv385, Btw375and385, Btw365and375, Below365*	Causative pathogen, current clinical phenotype, age group, respiratory symptoms	d16	Observable
b34	**Irritable**	Irritability in medical notes recorded during ED presentation.	*Yes, No*	Current clinical phenotype, age group, causative pathogen, temperature in ED	d16	Observable
b35	**Lethargy**	Lethargy in medical notes recorded during ED presentation.	*Yes, No*	Causative pathogen, current clinical phenotype, temperature in ED	d16	Observable
b36	**Nausea or vomiting**	Nausea and/or vomiting in medical notes recorded during ED presentation.	*Yes, No*	Causative pathogen, current clinical phenotype, age group	d16	Observable

vid	Variable label	Definition	States	Parents	Corresponding node/s in the Expert DAG v11	Status
b37	**Poor oral intake**	Poor oral intake in medical notes recorded during ED presentation.	*Yes, No*	Current clinical phenotype, age group, nausea or vomiting	d16	Observable

[1]Narrow: Amoxicilin, Trimethoprim, Benzylpenicillin, Cefalexin, Cefazolin, Erythromicin. Broader: Amoxicillin + Clavulanic acid, Trimethoprim + Sulfamethoxazole, Co-trimoxazole, Amikacin, Cefepime, Cefotaxime, Ceftazidime, Ceftriaxone, Ciprofloxacin, Colistin, Ertapenem, Gentamicin, Meropenem, Moxifloxacin, Nitrofurantoin, Norfloxacin, Piperacillin + Tazobactam, Tazocin, Tobramycin, Vancomycin

[2] This variable is latent, but was treated uniquely in order to provide a definition of current clinical phenotype that is independent of other latent factors in the model. In particular, a separate clustering was performed (using the EM algorithm) on the signs and symptoms, resulting in a grouping into three types, simply called “Type 1”, “Type 2” and “Type 3”, “Type 1” being systemic signs and symptoms predominant but mild urinary tract localising symptoms, “Type 2” being urinary tract localising symptoms predominant, and “Type 3” being abdominal pain predominant with minor other symptoms.
