# Supplementary material for: Urinary tract infections in children: building a causal model-based decision support tool for diagnosis with domain knowledge and prospective data
Source: BMC Med Res Methodol. 2022 Aug 8;22:218. doi: 10.1186/s12874-022-01695-6 (PMC9358867; doi:10.1186/s12874-022-01695-6)
Supplement: Supplementary file 6 — Additional file 6. Parameterisation survey responses. [file 12874_2022_1695_MOESM6_ESM.pdf]

Additional file 6: Parameterisation survey responses

In this document we provided a summary of survey responses used to inform the BN parameters.

Q1. Risk of specimen contamination

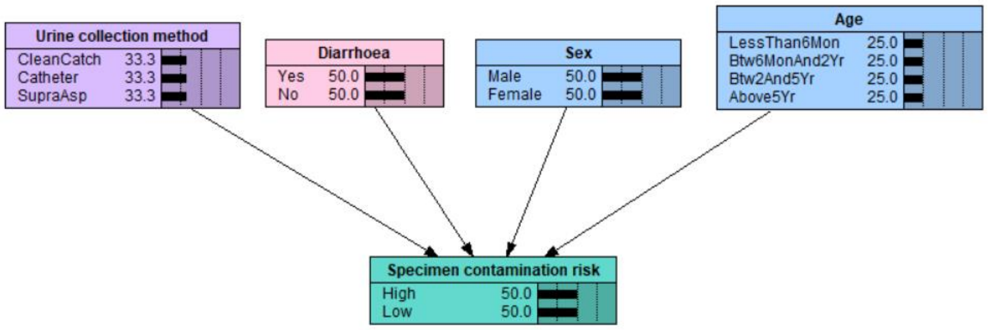

Consider the risk of a non-causative organism/s entering the urine specimen during the specimen collection process. In the model (as shown in the above figure), the **risk of specimen contamination** is influenced by **age**, **sex**, presence of **diarrhoea**, and **urine collection method**. Assuming *the same* colonisation status of each child's perineum/ external genitalia (i.e., type and density of organisms), how do the following factors increase or decrease the risk of specimen contamination from the baseline (as specified below)? E.g., x0.3, x2, x10, etc. Mean and standard deviation based on survey responses from 8 experts.

1a. **Age** and **sex**, assuming **clean catch** as the method of specimen collection.

**Age**	*Male*, mean (sd)	*Female*, mean (sd)
>=5yo	1 (baseline)	1.8 (1.0)
2 to 5yo	2.2 (0.7)	3.6 (2.0)
6mon to 2yo	3.3 (1.4)	5.2 (3.1)
<6mon	4.2 (2.1)	6.6 (4.0)

1b. Presence of **diarrhoea**, assuming **clean catch** as the method of specimen collection.

Absence	1 (baseline), mean (sd)
Presence	4.1 (1.6)

1c. **Urine collection method**

Supra aspirate	1 (baseline), mean (sd)
Catheter	2.3 (1.2)
Clean catch	6.8 (5.4)

Collated comments

2-5 year old age group above would also be influenced by whether the child is toilet trained or not as yet.
the organisms introduced relate more to how clean a catch it is…if that makes any sense. Older kids pass urine straight into the cup, can do mid-stream when older again. A small child will be wiped clean but then gets progressively dirty waiting for the catch and often the urine sprays everywhere, has touched skin on way through.
If female with diarrhoea is at highest risk.
Could consider circumcision in boys reducing the contamination rates
https://pubmed.ncbi.nlm.nih.gov/22537082/

Q2. Propensity to UTI progression

Consider a child's risk of progressing to more severe disease manifestations given they have a UTI, e.g., developing kidney infection, or experiencing worsening severity of local or systemic inflammatory response, which can be further broken into two concepts: the **speed of progression**, and the **susceptibility to severity** – illustrated using the diagram below. Please note that these curves are illustrative, not exact.

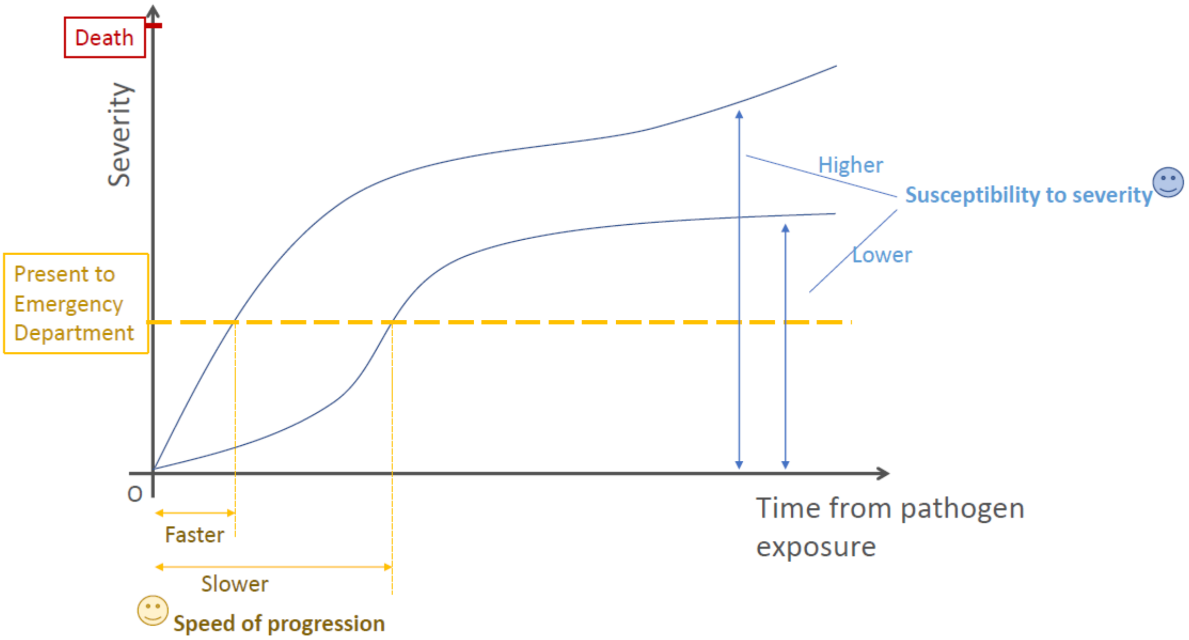

In the model, both the **speed of progression** and **susceptibility to severity** may be influenced by **age** and **UTI-relevant comorbidity** (such as VUR/anatomical abnormalities of the urinary tract). We now ask a series of questions on these two concept variables. Please provide your min, max, and best guess estimates for each question. Please note that the "min/max" should be plausible lower or upper values, e.g., 95th percentiles, not the extreme recordable value. Mean and standard deviation based on survey responses from 8 experts.

2a. **Speed of progression**

Assuming a baseline speed of progression (as specified below), how do the following factors increase or decrease the baseline? E.g., x0.3, x2, x10, etc.

Age

>=5yo	Speed of progression = 1 (baseline)		
**Estimate**	**Min**, mean	**Max**, mean	**Best**, mean (sd)
2 to 5yo	0.9	3.9	1.8 (0.7)
6mon to 2yo	1.2	4.9	3.2 (1.8)
<6mon	1.8	7.4	5.5 (3.6)

UTI-relevant comorbidity

No comorbidity	Speed of progression = 1 (baseline)		
**Estimate**	**Min**, mean	**Max**, mean	**Best**, mean (sd)
With comorbidity	1.3	6.1	3.3 (2.0)

2b. **Susceptibility to severity**

Assuming a baseline susceptibility to severity (as specified below), how do the following factors increase or decrease the baseline? E.g., x0.3, x2, x10, etc.

Age

>=5yo	Susceptibility to severity = 1 (baseline)		
**Estimate**	**Min**, mean	**Max**, mean	**Best**, mean (sd)
2 to 5yo	1.0	4.1	1.7 (0.8)
6mon to 2yo	1.2	4.8	3.0 (2.0)
<6mon	1.7	6.3	5.1 (3.9)

UTI-relevant comorbidity

No comorbidity	Susceptibility to severity = 1 (baseline)		
**Estimate**	**Min**, mean	**Max**, mean	**Best**, mean (sd)
With comorbidity	1.4	6.3	3.6 (1.8)

Collated comments

I would categorise age <1mo as much higher risk than 1-5 months.<br>Many congenital renal abnormalities would not necessarily predispose to a more rapid progression (eg VUR Grade 1-III), but some would definitely increase risk of more rapid progression to disease (eg posterior urethral valves in a child <6mo)
(Speed of progression) Pathogens particularly virulent e.g K1 E coli, presence of comorbidity and a foreign body may play a role such as the presence of a stent
(Susceptibitlity to severity) Just thinking of some patients that don't follow these trends – not sure my clinician knowledge is that good when you think about all the patients that don't fit the text book!!
(Speed of progression) Will be affected by immunosuppression.<br>Is impacted by congenital bladder and renal tract anomalies. However, this is hard to quantify on a baseline of an increased frequency of infections, many of which do not progress past cystitis.
(Susceptibitlity to severity) This is on a low baseline risk.
Different comorbidities likely to affect speed of progression.
Lumping co-morbidities in one may be misleading – mild degree of VUR is unlikely to make a difference, but severe VUR will
Severe and progression I think are likely to be interrelated

Q3. Causative pathogen for UTI

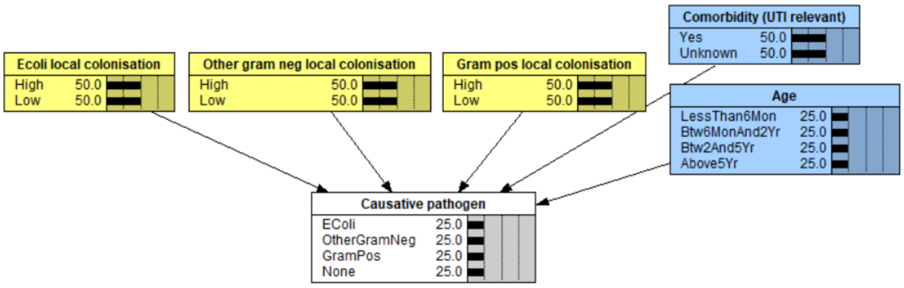

Colonisation of the perineum/ external genitalia by bacteria is assumed to predispose children to urinary tract infection (UTI). In the model, the probability of UTI with each causative pathogen (shown as **causative pathogen** in the above figure) is influenced by **age**, **local colonisation**, and **UTI-relevant comorbidity** (such as VUR/anatomical abnormalities of the urinary tract).

3a. Consider the PEA cohort, we enrolled children who presented to the Emergency Department (ED) at Perth Children's Hospital and were managed for presumed UTI (with an antibiotic prescription in the ED and a urine sample sent for laboratory investigation). These patients typically underwent urine dipstick in the ED. What do you estimate the probability (min, max, best guess) of true UTI in this cohort (prior to seeing the laboratory culture result)? Please note that the "min/max" should be plausible lower or upper values, not the extreme recordable value. Mean and standard deviation based on survey responses from 8 experts.

	True UTI, in %					
**Age**	*Boy*			*Girl*		
**Estimate**	**Min,** mean	**Max,** mean	**Best**, mean (sd)	**Min,** mean	**Max,** mean	**Best**, mean (sd)
>=5yo	59	83	77 (30)	58	88	76 (19)
2 to 5yo	54	92	70 (20)	53	92	73 (12)
6mon to 2yo	54	91	61 (28)	52	89	63 (24)
<6mon	54	86	62 (29)	51	84	63 (29)

Collated comments

Even with a culture positive, sometimes the dipstick/microscopy/clinical interpretation is suboptimal. In see a lot of children with recurrent UTI in clinic, in maybe 20% of these referrals the children are mostly having misdiagnosed infections (i.e. contamination labelled as UTI). Colonisation is a urine consistent with UTI, but no symptoms and this is VERY hard to model, and poorly understood by paediatricians (now widely accepted in adult medicine).<br><br>The probability of a positive culture after Rx for UTI in ED will be inflated by contamination.
I am assuming that that a prescription would be driven by an abnormal UA, so this question is what is the likelihood of a true UTI in the presence of an abnormal urinalysis.<br><br>The sensitivity and specificity of different components of urinalysis vary which makes estimating these numbers difficulty<br><br>Although tradition suggests that sensitivity and specificity varies by age, this is in my mind, overstated and that it is remains a good test in young children

3b. In the case of an otherwise healthy child with colonisation of the perineum/ external genitalia by all the following three groups of organisms: E.coli, other gram negatives, and gram positives. Note,

we refer to gram positives that can potentially cause UTI, such as Enterococcus, rather than gram positives like Staph epidermidis which are unlikely cause UTI. Mean and standard deviation based on survey responses from 8 experts.

	Consider the E coli pathogenicity as baseline, could you please indicate the relative pathogenicity of others? E.g., x0.5, x3, etc., mean (sd)	Please comment if different pathogens affect the **speed of progression** and **susceptibility to severity** differently? If so, how?
E.coli	1 (baseline)	
Other gram negatives	1.35 (0.53)	
Gram positives (e.g., Enterococcus)	0.98 (0.91)	

Collated comments

GramPos: Might be less than gram negatives, but do not have a good feel for this.
OtherGramNeg: Variable speed and severity (likely potentially lower, dependent on bug e.g., Kleb similar but Serratia lower)
GramPos: Low speed and severity
OtherGramNeg: Pseudomonas 2, Resistant gram negatives e.g ESBL 3; Particularly in a younger child, Given less likely to have early effective treatment and more challenging to treat
GramPos: Less severe and progressive
OtherGramNeg: Potentially more rapid spread with other gram negatives in the context of underlying renal anomalies and particularly with those with a stent in situ (biofilm formation, eg with organisms such as Pseudomonas aeruginosa).
GramPos: Generally less likely to cause disseminated infection.
E coli more frequently coloniseds, but the pathogenicity of this compared with other Enterobactericeae (e.g. Klebsiella, Enterobacter) is the same. Enterococci are the weeds of the urinary tract – in regard to pathogenicity, speed and severity

Q4. Impact of exiting antibiotic use

4a. For modelling purpose, we have grouped antibiotics into two groups: narrow and broader, could you please review this grouping and suggest if any antibiotic should be grouped differently? Please feel free to add new group/s.

Narrow: Amoxicilin, Amoxicillin + clavulanic acid, Trimethoprim, Trimethoprim + Sulfamethoxazole, Benzylpenicillin, Cefalexin, Cefazolin, Co-trimoxazole, Erythromicin

Broader: Amikacin, Cefepime, Cefotaxime, Ceftazidime, Ceftriaxone, Ciprofloxacin, Colistin, Ertapenem, Gentamicin, Meropenem, Moxifloxacin, Nitrofurantoin, Norfloxacin, Piperacillin + Tazobactam, Tazocin, Tobramycin, Vancomycin

Comment: From only one expert.

Please see attached for a published list of antibiotics with their proposed “spectrum score”. Thus, I’d suggest you compare your below two groupings to ensure that all those in the “narrow” group have spectrum scores lower than all those in the “broader” group. Based on this you may need to move a few antibiotics between the two groups to ensure they are grouped appropriately (eg Amoxicillin + clavulanic acid and Co-trimoxazole probably need to be in the “broader” group) . This would have the additional benefit of being able to reference your classification.

4b. Given a UTI, the successful detection of the causative pathogen of the UTI in laboratory can be influenced if the patient has been on antibiotic when they came to the ED where the urine sample was taken. Presumably this is largely affected by the antimicrobial susceptibility pattern of the pathogen which can variable by different subgroups, so please consider an average community-acquired case in 2019-2020.

Under the following scenarios, please provide your min, max, and best guess estimates for each question. Please note that the "min/max" should be plausible lower or upper values, not the extreme recordable value. (Please feel free to refer to your experience of treating UTI in adults.) Response received from only one expert.

Consider a UTI caused by E.coli

	Probability of positive culture of E.coli		
**Estimate**	**Min**	**Max**	**Best guess**
Not on abx	50	100	80
On narrow abx	20	50	30
On broader abx	10	30	20
Pls feel free to add more groups			

Consider a UTI caused by other gram negative bacteria

	Probability of positive culture of other gram neg		
**Estimate**	**Min**	**Max**	**Best guess**
Not on abx	50	100	80
On narrow abx	20	50	30
On broader abx	10	30	20

Consider a UTI caused by gram positive bacteria (e.g., Enterococcus)

	Probability of positive culture of gram pos		
**Estimate**	**Min**	**Max**	**Best guess**
Not on abx	50	100	80
On narrow abx	20	50	30
On broader abx	10	30	20

Comment:

In the absence of any data to support my estimates, my guesses are the same for E.coli/gram neg/gram positives.
